# Blocking interaction of sclerostin loop3 with osteoblastic LRP4 counteracts bone loss without increasing arterial stiffness during mechanical unloading

**DOI:** 10.1016/j.jot.2026.101117

**Published:** 2026-06-08

**Authors:** Luyao Wang, Ning Zhang, Hewen Jiang, Xiaohui Tao, Yuan Ma, Zebing Hu, Chuanxin Zhong, Xin Yang, Sifan Yu, Huarui Zhang, Jin Liu, Yuanyuan Yu, Fangwu Liu, Zefeng Chen, Hang Luo, Shijian Ding, Yihao Zhang, Meiheng Sun, Shiqing Zhang, Péter Ferdinandy, Rongjun Yu, Tao Zhang, Aiping Lu, Ling Qin, Shu Zhang, Baoting Zhang, Ge Zhang

**Affiliations:** a Law Sau Fai Institute for Advancing Translational Medicine in Bone and Joint Diseases (TMBJ), Hong Kong Baptist University, Hong Kong SAR, China; b Guangdong-Hong Kong-Macao Greater Bay Area International Research Platform for Aptamer-based Translational Medicine and Drug Discovery (HKAP), Hong Kong SAR, China; c Institute of Integrated Bioinformedicine and Translational Science (IBTS), Hong Kong Baptist University, Hong Kong SAR, China; d School of Chinese Medicine, Faculty of Medicine, The Chinese University of Hong Kong, Hong Kong SAR, China; e The Key Laboratory of Aerospace Medicine, Ministry of Education, Air Force Medical University, Xi'an, Shaanxi, China; f Shanghai Institute of Technical Physics Chinese Academy of Sciences, Shanghai, China; g State Key Laboratory of Bioactive Molecules and Druggability Assessment, Jinan University, Guangzhou, China; h Department of Pharmacology and Pharmacotherapy, Semmelweis University, Budapest, Hungary; i Academy of Wellness and Human Development, Hong Kong Baptist University, Hong Kong SAR, China

## Abstract

**Background:**

Mechanical unloading leads to bone loss and cardiovascular deconditioning, accompanied by elevated sclerostin expression. Genetic *Sost* knockout or pharmacologic sclerostin antibody treatment was reported to counteract bone loss during mechanical unloading in mice. However, severe cardiovascular events were reported in postmenopausal osteoporotic patients treated with commercially available sclerostin antibody targeting loop2. It is desirable to develop a precise sclerostin inhibition strategy to counteract unloading-induced bone loss, without increasing cardiovascular risk.

**Methods and results:**

In a previously published rodent studies under normal loading condition, it was found that sclerostin loop3 participated in the inhibitory effect of sclerostin on bone formation, while the preventive action of sclerostin against cardiovascular events was independent of sclerostin loop3. Nevertheless, whether and how sclerostin loop3 contributes to bone formation reduction and bone loss under mechanical unloading condition remains unclear. In this study under mechanical unloading condition, either sclerostin loop3-specific deficiency in *Sost*^*loop3−/*^^−^ mice or sclerostin loop3-specific inhibition by our tailor-made aptamer Apc001 counteracted unloading-induced bone loss without increasing arterial stiffness, whereas either *Sost* knockout or romosozumab treatment significantly increased unloading-induced arterial stiffness in mice. These findings indicated sclerostin loop3 as a therapeutic target with cardiovascular safety against unloading-induced bone loss. Mechanistically, we identified that sclerostin loop3 bound to LRP4 in osteoblasts under mechanical unloading condition. Osteoblast-specific *Lrp4* knockout counteracted unloading-induced bone formation reduction and bone loss in *OB. Lrp4*^*−/−*^ mice. Further, blocking the interaction of sclerostin loop3 with LRP4 via mutation of the interaction residues (*Lrp4m*) or pharmacologic inhibition with LRP4 peptide tool (LRP4-Pep) dramatically attenuated binding of sclerostin to LRP6, counteracted decrease of Wnt/β-catenin signaling activity and osteogenic potential in osteoblasts under mechanical unloading condition *in vitro*. Consistently, *Lrp4m* counteracted unloading-induced bone formation reduction and bone loss in mice *in vivo*. In *Lrp4m/OB-Lrp4* mice, osteoblast-conditional correction of *Lrp4m* to wild-type *Lrp4* attenuated the counteractive effect of *Lrp4m* on unloading-induced bone loss. Pharmacologically, osteoblasts-targeted LRP4-Pep counteracted bone formation reduction and bone loss during mechanical unloading in wild-type mice.

**Conclusion:**

Sclerostin loop3-mediated anchoring of sclerostin to LRP4 facilitated its binding to LRP6 in osteoblasts, contributing to bone formation reduction and bone loss under mechanical unloading condition.

**The translational potential of this article:**

Specifically blocking the interaction of sclerostin loop3 with LRP4 in osteoblasts would offer a precise strategy with cardiovascular safety for treatment of unloading-induced bone loss.

## Graphical abstract

**Figure:**
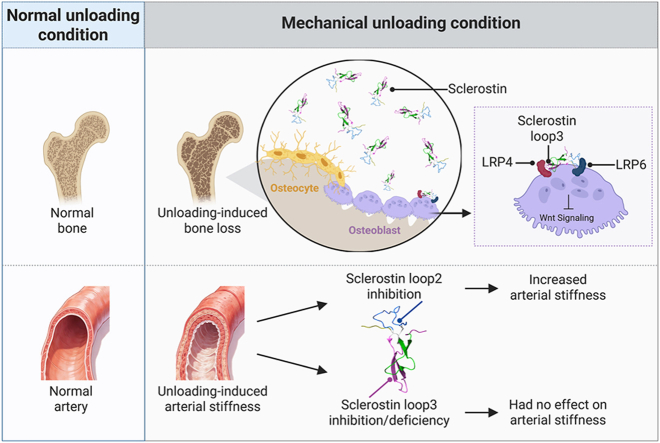

## 1 Introduction

Mechanical unloading, whether from prolonged bed rest or microgravity, leads to significant bone loss through bone remodeling imbalance characterized by reduced formation and increased resorption [1,2]. Bisphosphonates are first-line antiresorptive agents that inhibit osteoclast-mediated bone resorption and are widely used to treat osteoporosis. However, it has been reported that high doses of bisphosphonates showed poor efficacy in preserving bone mass and strength in unloading-induced bone loss [3,4]. It is important to investigate potential therapeutic strategies to promote bone formation and counteract unloading-induced bone loss.

Sclerostin is a negative regulator of bone formation, which is sensitive to mechanical forces [5]. It was reported that mechanical unloading caused rapid release of sclerostin from osteocytes [6]. Serum sclerostin levels were reported to be significantly elevated in both bedridden patients and astronauts, while skeletal *Sost* mRNA expression levels were dramatically increased in mice after spaceflight [[7], [8], [9], [10], [11], [12], [13]]. It was consistent with the results of ground-based studies in rodents with hindlimb unloading [7,12,14]. Genetic *Sost* knockout or pharmacologic sclerostin antibody treatment was reported to counteract bone formation reduction and bone loss in mice with hindlimb unloading [12,[14], [15], [16]]. Collectively, sclerostin could be a therapeutic target for unloading-induced bone loss. However, severe cardiovascular events were reported with therapeutic sclerostin antibody (romosozumab) in postmenopausal osteoporotic patients [17,18]. Romosozumab for postmenopausal osteoporosis was approved by US-FDA with an one-year use restriction and a black-boxed warning on the risk of stroke and cardiovascular death (FDA Press Announcements). In addition, the cardiovascular system of bedridden patients was reported to adapt to immobilization with significant deconditioning [19,20]. Cardiovascular monitoring data from astronauts on the International Space Station showed a significant increase in their arterial stiffness, which was equivalent to 10-20 years of aging changes in general populations on the ground [21]. Furthermore, we found that romosozumab significantly increased arterial stiffness of mice with hindlimb unloading [22]. Thus, for bedridden patients and astronauts, the increased cardiovascular risk of sclerostin inhibition cannot be excluded. It is desirable to develop a precise sclerostin inhibition strategy to counteract unloading-induced bone loss, without increasing cardiovascular risk.

The central residues of sclerostin form three loops, including loop1, loop2 and loop3 [23]. Humanized therapeutic sclerostin antibody (romosozumab) mainly targets sclerostin loop2 [24]. In a previously published rodent studies under normal loading condition, it was notably found that sclerostin loop3 participated in the antagonistic effect of sclerostin on bone formation, while the preventive action of sclerostin on cardiovascular events was independent of loop3. Specifically targeting sclerostin loop3 by our tailor-made aptamer Apc001 (IC_50_ = 19.7 μg/mL) promoted bone formation, increased bone mass and enhanced bone strength in both ovariectomized rats and *Col1a2*^*+/G610C*^ mice, without increasing cardiovascular risk [25,26]. The aptamer Apc001 with cardiovascular safety was granted Orphan-Drug Designation and Rare-Pediatric-Disease Designation by US-FDA for treatment of osteogenesis imperfecta. Currently, Apc001 has completed Pilot-Scale Production Stage. Sclerostin loop3 emerges as a precise bone anabolic target with cardiovascular safety under normal loading condition.

Further in this study, Apc001 against sclerostin loop3 counteracted unloading-induced bone loss in mice without increasing arterial stiffness. It was consistent with the effects of sclerostin loop3 deficiency in *Sost*^*loop3−/*^^*−*^ mice with hindlimb unloading. In contrast, the antibody against sclerostin loop2 significantly increased arterial stiffness. Accordingly, sclerostin loop3 could be a target with cardiovascular safety against mechanical unloading-induced bone loss. Mechanistically, how sclerostin loop3 contributes to bone loss under mechanical unloading condition warrants investigation. We hypothesized that sclerostin loop3-mediated anchoring of sclerostin to LRP4 could facilitate its binding to LRP6 in osteoblasts, contributing to bone formation reduction and bone loss under mechanical unloading condition. It would pave the way for developing a precise therapeutic strategy for unloading-induced bone loss without increasing arterial stiffness.

## 2 Results

### 2.1 Either sclerostin loop3-specific deficiency in *Sost*^*loop3−/*^^*−*^ mice or sclerostin loop3-specific inhibition by our tailor-made aptamer Apc001 counteracted unloading-induced bone formation reduction and bone loss without increasing arterial stiffness

The hindlimb unloading mouse model, established via tail suspension, was a well-accepted and widely used model for studying the effects of mechanical unloading on bone formation and arterial stiffness [27]. To determine whether sclerostin loop3 could contribute to unloading-induced bone formation reduction and bone loss*,* the bone mass, bone microarchitecture, bone formation and bone mechanical properties were determined in wild-type (WT) mice, *Sost*^*−/−*^ mice and *Sost*^*loop3−/*^^*−*^ mice, with/without hindlimb unloading for four weeks. Translationally, the counteractive effects of our tailor-made sclerostin loop3-specific aptamer Apc001 on bone formation reduction and bone loss were determined in WT mice, following hindlimb unloading for four weeks (Fig. S1).

**Fig. 1:**
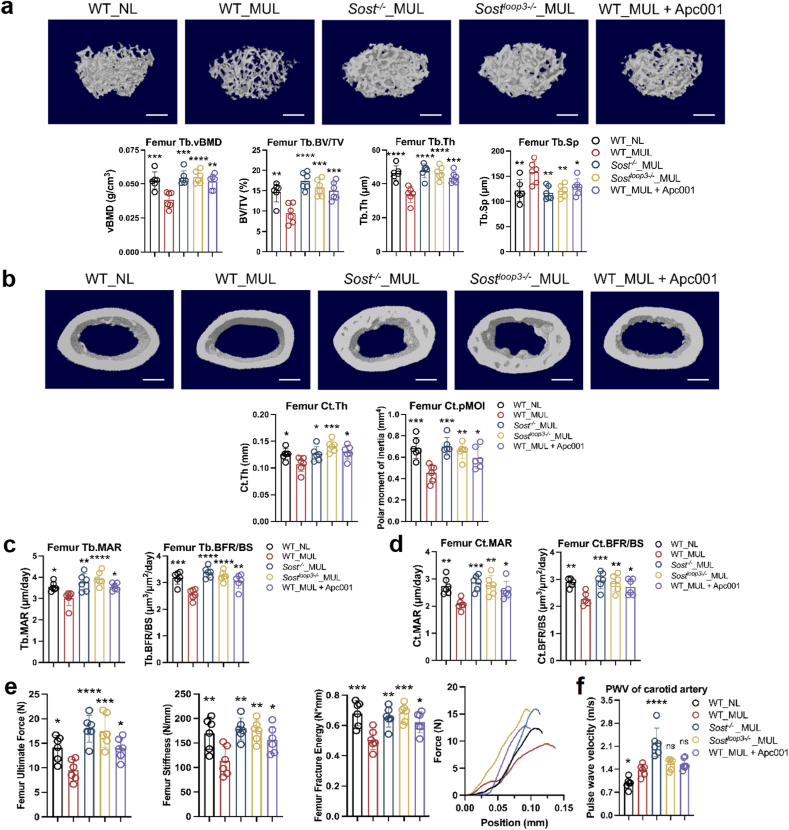
Either sclerostin loop3-specific deficiency in *Sost*^*loop3−/*^^*−*^ mice or sclerostin loop3-specific inhibition by Apc001 counteracted unloading-induced bone formation reduction and bone loss without increasing arterial stiffness. **(a)** Representative images showing three-dimensional trabecular architecture by micro-CT reconstruction at distal femur of mice (the upper panel). Scale bars, 200 μm. Bar charts of Tb.vBMD, Tb.BV/TV, Tb.Th and Tb.Sp (the lower panel). **(b)** Representative images showing three-dimensional cortical bone architecture by micro-CT reconstruction at femoral mid-shaft (the upper panel). Scale bars, 200 μm. Bar charts of Ct.Th and Ct.pMOI (the lower panel). **(c)** Dynamic bone histomorphometric parameters of Tb.MAR and Tb. BFR/BS at distal femur. **(d)** Dynamic bone histomorphometric parameters of Ct.MAR and Ct.BFR/BS at femoral mid-shaft. **(e)** Three-point bending test for femur ultimate force (left), femur stiffness (middle left), and femur fracture energy (middle right). Representative curves showing the mechanical properties of femur (right). **(f)** Bar chart of carotid artery stiffness, determined by doppler ultrasound analysis. Data were expressed as mean ± standard deviation. N = 6 per group. ^ns^ P > 0.05, ∗P < 0.05, ∗∗P < 0.01, ∗∗∗P < 0.001, ∗∗∗∗P < 0.0001 for a comparison vs. WT_MUL by one-way ANOVA with Tukey's post-hoc test. **NOTE**: NL: normal loading; MUL: mechanical unloading; Tb.vBMD: trabecular volumetric bone mineral density, Tb.BV/TV: trabecular bone volume per total volume; Tb.Th: trabecular thickness; Tb.Sp: trabecular spacing; Ct.Th: cortical thickness; Ct.pMOI: cortical polar moment of inertia; Tb.MAR: trabecular bone mineral apposition rate; Tb.BFR/BS: trabecular bone formation rate; Ct.MAR: cortical bone mineral apposition rate; Ct.BFR/BS: cortical bone formation rate; PWV: pulse wave velocity.

Micro-CT analysis was used to determine bone mass and bone microarchitecture at trabecular bone of distal femur and proximal tibia, as well as at cortical bone of femoral mid-shaft. The trabecular volumetric bone mineral density (Tb.vBMD), trabecular relative bone volume per total volume (Tb.BV/TV), and trabecular thickness (Tb.Th) were significantly lower, while trabecular spacing (Tb.Sp) was significantly higher in WT_MUL (mechanical unloading) mice than those in WT_NL (normal loading) mice at the above three sites (Fig. 1&S2), indicating unloading-induced bone loss was established with substantially lower trabecular bone mass and worse trabecular bone microarchitecture in mice with hindlimb unloading. In comparison, both *Sost*^*−/−*^_MUL mice and *Sost*^*loop3−/*^^−^_MUL mice had significantly higher Tb.vBMD, Tb.BV/TV, Tb.Th, and lower Tb.Sp at trabecular bone of distal femur (Fig. 1a), as well as higher Tb.vBMD, Tb.BV/TV, Tb.Th, and lower Tb.Sp at trabecular bone of proximal tibia (Fig. S2c) than WT_MUL mice. After Apc001 treatment, the Tb.vBMD, Tb.BV/TV and Tb.Th at both trabecular bone of distal femur and trabecular bone of proximal tibia were significantly enhanced in WT_MUL mice, while Tb. Sp at both trabecular bone of distal femur and trabecular bone of proximal tibia were significantly decreased (Fig. 1a&S2c). Compared to WT_NL mice, the cortical thickness (Ct.Th) and cortical polar moment of inertia (Ct.pMOI) at cortical bone of femoral mid-shaft were significantly lower in WT_MUL mice (Fig. 1b), indicating that bone loss was established with substantially lower cortical bone thickness and worse cortical bone microarchitecture in mice with hindlimb unloading. In contrast, both *Sost*^*−/−*^_MUL mice and *Sost*^*loop3−/*^^−^_MUL mice had significantly higher Ct.Th and Ct.pMOI at cortical bone of femoral mid-shaft than WT_MUL mice (Fig. 1b). After Apc001 treatment, the Ct.Th and Ct.pMOI at cortical bone of femoral mid-shaft were significantly enhanced in WT_MUL mice (Fig. 1b).

Consistently, bone histomorphometric analysis was used to determine the bone formation at trabecular bone of distal femur and proximal tibia, as well as at cortical bone of femoral mid-shaft. The data showed that WT_MUL mice had significantly lower trabecular bone mineral apposition rate (Tb.MAR) and lower trabecular bone formation rate (Tb.BFR/BS) at trabecular bone of distal femur and proximal tibia (Fig. 1c&S2a&S2d), as well as significantly lower cortical bone mineral apposition rate (Ct.MAR) and lower cortical bone formation rate (Ct.BFR/BS) at cortical bone of femoral mid-shaft than WT_NL mice (Fig. 1d&S2b). Both *Sost*^*−/−*^_MUL mice and *Sost*^*loop3−/*^^*−*^_MUL mice had significantly higher Tb.MAR and Tb.BFR/BS at trabecular bone of distal femur (Fig. 1c&S2a), as well as higher Tb.MAR and Tb. BFR/BS at trabecular bone of proximal tibia than WT_MUL mice (Fig. S2d). After Apc001 treatment, the Tb.MAR and Tb.BFR/BS at trabecular bone of both distal femur and proximal tibia were significantly enhanced in WT_MUL mice (Fig. 1c&S2a&S2d). Moreover, both *Sost*^*−/−*^_MUL mice and *Sost*^*loop3−/*^^*−*^_MUL mice had significantly higher Ct.MAR and Ct.BFR/BS at cortical bone of femoral mid-shaft than WT_MUL mice (Fig. 1d&S2b). After Apc001 treatment, the Ct.MAR and Ct.BFR/BS at cortical bone of femoral mid-shaft were significantly enhanced in WT_MUL mice (Fig. 1d&S2b).

Further, three point bending test was used to determine the bone mechanical properties of femur. The femur ultimate force, femur stiffness and femur fracture energy were significantly higher in both *Sost*^*−/−*^_MUL mice and *Sost*^*loop3−/*^^*−*^_MUL mice, when compared to those in WT_MUL mice. After Apc001 treatment, the femur ultimate force, femur stiffness and femur fracture energy were significantly increased in WT_MUL mice (Fig. 1e).

In doppler ultrasound analysis [28], WT_MUL mice had significantly higher arterial Pulse Wave Velocity (PWV) than WT_NL mice. *Sost*^*−/−*^_MUL mice had significantly higher arterial PWV than WT_MUL mice. There was no significant difference in arterial PWV between *Sost*^*loop3−/*^^*−*^_MUL mice and WT_MUL mice. Moreover, there was no significant difference in arterial PWV of WT_MUL mice between with and without Apc001 treatment (Fig. 1f). In contrast, the arterial PWV was significantly enhanced in WT_MUL mice after sclerostin antibody (romosozumab) treatment (Fig. S2e).

Either sclerostin loop3-specific deficiency or sclerostin loop3-specific inhibition by Apc001 counteracted unloading-induced bone loss without increasing arterial stiffness, whereas either *Sost* knockout or sclerostin antibody treatment significantly increased unloading-induced arterial stiffness in mice. **Sclerostin loop3 could be a therapeutic target with cardiovascular safety against unloading-induced bone loss.** Nevertheless, how sclerostin loop3 contributes to bone formation reduction and bone loss under mechanical unloading condition remains unclear.

### 2.2 Osteoblast-specific *Lrp4* knockout counteracted unloading-induced bone formation reduction and bone loss in *OB*.*Lrp4*^*−/−*^ mice

**Fig. 2:**
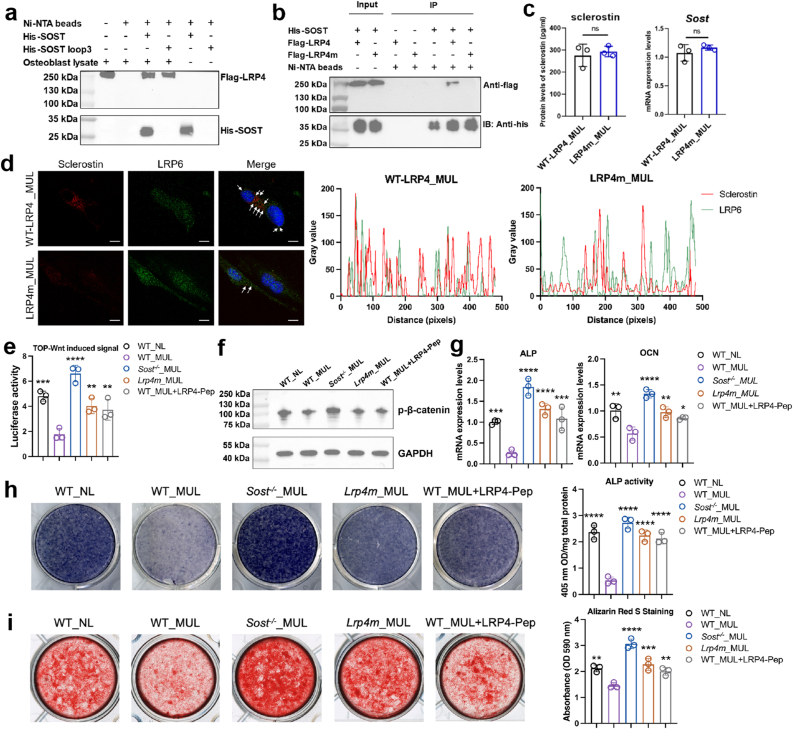
Blocking interaction of sclerostin loop3 with LRP4 attenuated binding of sclerostin to LRP6, counteracted decrease of Wnt/β-catenin signaling activity and osteogenic potential in osteoblasts under mechanical unloading condition *in vitro*. **(a)** Binding analysis for the interaction of LRP4 to sclerostin (His-SOST) and sclerostin loop3 (His-SOST loop3) in osteoblasts (MC3T3-E1 cells) by pull-down assay under mechanical unloading (MUL) condition. **(b)** The binding ability of LRP4 and LRP4m to sclerostin (His-SOST) in MC3T3-E1 cells by co-IP assay under MUL condition. **(c)** The protein levels of sclerostin in MC3T3-E1 cells (Lysate and medium) under MUL condition, determined by ELISA (the left panel). The mRNA expression of *Sost* in MC3T3-E1 cells under MUL condition, determined by RT-qPCR (the right panel). **(d)** Confocal immunomicroscopic analysis of MC3T3-E1 cells under MUL condition (the left panel). The MC3T3-E1 cells were probed for sclerostin (Red fluorescence) and LRP6 (Green fluorescence). Arrows indicated the co-location of sclerostin and LRP6. Scale bars, 10 μm. Offset white lines indicating intensity traces (the right panel). **(e)** The Wnt/β-catenin signaling in MC3T3-E1 cells incubated with the conditioned medium from MLO-Y4 cells under MUL condition. **(f)** The protein level of p-β-catenin in MC3T3-E1 cells incubated with the conditioned medium from MLO-Y4 cells under MUL condition. **(g)** The mRNA expression of ALP and OCN in MC3T3-E1 cells incubated with the conditioned medium from MLO-Y4 cells under MUL condition. **(h)** ALP activity staining (the left panel) and bar chart of ALP activity (the right panel) in MC3T3-E1 cells incubated with the conditioned medium from MLO-Y4 cells under MUL condition. **(i)** Alizarin Red S Staining (the left panel) and bar chart of the mineralized nodule formation (the right panel) in MC3T3-E1 cells incubated with the conditioned medium from MLO-Y4 cells under MUL condition. Data were expressed as mean ± standard deviation. N = 3 per group. ^ns^ P > 0.05, ∗P < 0.05, ∗∗P < 0.01, ∗∗∗P < 0.001, ∗∗∗∗P < 0.0001 for a comparison vs. WT_MUL by one-way ANOVA with Tukey's post-hoc test. **NOTE**: NL: normal loading; MUL: mechanical unloading; ALP: alkaline phosphatase; OCN: osteocalcin; LRP4-Pep: LRP4 peptide tool; p-β-catenin: phosphate β-catenin.

In our study, sclerostin loop3 was found to bind to LRP4 rather than to LRP6 in osteoblasts under normal loading condition [29]. Consistently under mechanical unloading condition, sclerostin loop3 bound to LRP4 in osteoblasts in pull-down assay (Fig. 2a). LRP4 in osteoblasts was reported to participate in inhibiting bone formation under normal loading condition [30,31]. To determine the role of osteoblastic LRP4 in bone under mechanical unloading condition, we generated osteoblast-specific *Lrp4* knockout (*OB*.*Lrp4*^*−/−*^) mouse model. Then, the bone mass, bone microarchitecture, bone formation and bone mechanical properties were determined in WT mice and *OB*.*Lrp4*^*−/−*^ mice, with/without hindlimb unloading for four weeks (Fig. S3a).

In *micro-CT* analysis, when compared to WT mice under normal loading condition (WT_NL), WT mice under mechanical unloading condition (WT_MUL) had significantly lower Tb.vBMD, Tb.BV/TV, Tb.Th and higher Tb.Sp at trabecular bone of distal femur, as well as significantly lower Ct. Th and Ct. pMOI at cortical bone of femoral mid-shaft (Fig. S3b&S3d). The *OB.Lrp4*^*−/−*^ mice under mechanical unloading condition (*OB.Lrp4*^*−/−*^*_*MUL) had significantly higher Tb.vBMD, Tb.BV/TV, Tb.Th and lower Tb.Sp at trabecular bone of distal femur, as well as significantly higher Ct.Th and Ct.pMOI at cortical bone of femoral mid-shaft than WT mice under mechanical unloading condition (WT_MUL) (Fig. S3b&S3d).

In bone histomorphometric analysis, Tb.MAR and Tb.BFR/BS at trabecular bone of distal femur, as well as Ct.MAR and Ct.BFR/BS at cortical bone of femoral mid-shaft were significantly lower in WT_MUL mice, when compared to WT_NL mice (Fig. S3c&S3e). The *OB.Lrp4*^*−/−*^*_*MUL mice had significantly higher Tb.MAR and Tb.BFR/BS at trabecular bone of distal femur, as well as higher Ct.MAR and Ct.BFR/BS at cortical bone of femoral mid-shaft than WT_MUL mice (Fig. S3c&S3e).

In three-point bending test at femora, the femur ultimate force, femur stiffness and femur fracture energy were significantly lower in WT_MUL mice than those in WT_NL mice (Fig. S3f). The *OB.Lrp4*^*−/−*^*_*MUL mice had significantly higher femur ultimate force, femur stiffness and femur fracture energy than WT_MUL mice (Fig. S3f).

The above data indicated that osteoblast-specific *Lrp4* knockout counteracted unloading-induced bone formation reduction and bone loss in *OB*.*Lrp4*^*−/−*^ mice with hindlimb unloading.

### 2.3 Blocking interaction of sclerostin loop3 with LRP4 attenuated binding of sclerostin to LRP6, counteracted decrease of Wnt/β-catenin signaling activity and osteogenic potential in osteoblasts under mechanical unloading condition *in vitro*

Compared to MLO-Y4 cells and MC3T3-E1 cells under normal loading condition, the mRNA/protein expression of *Sost*/sclerostin were significantly higher in MLO-Y4 cells and MC3T3-E1 cells under mechanical unloading condition (Fig. S4). In the functional studies, we used the conditional medium of osteocytes (MLO-Y4 cells) to culture osteoblasts (MC3T3-E1 cells) under normal loading or mechanical unloading condition. The WT MC3T3-E1 cells cultured in WT MLO-Y4 cell medium under mechanical unloading condition (WT_MUL) showed significantly lower Wnt/β-catenin signaling, osteogenic potential and less mineralized nodule formation than the WT MC3T3-E1 cells cultured in WT MLO-Y4 cell medium under normal loading condition (WT_NL) (Fig. 2e–i). The *Sost*^*−/−*^ MC3T3-E1 cells cultured in *Sost*^*−/−*^ MLO-Y4 cell medium under mechanical unloading condition (*Sost*^*−/−*^_MUL) had significantly higher Wnt/β-catenin signaling, osteogenic potential and more mineralized nodule formation than the WT MC3T3-E1 cells cultured in WT MLO-Y4 cell medium under mechanical unloading condition (WT_MUL).

We identified the LRP4 residues (Y200, G201, L205, D206, I207, Y208, H209, C210) that interacted with sclerostin loop3 [32]. Then, *Lrp4* mutation tool (*Lrp4m*, encoding LRP4m-Y200A/G201A/Y208A/H209A/C210A) and osteoblasts-targeted LRP4 blocking peptide tool (LRP4-Pep, an LRP4 LA5 fragment conjugated to the bone-formation-surface-targeting oligopeptide (DSS)_6_) [33,34] were engineered to specifically block the interaction of sclerostin loop3 with LRP4, without altering Wnt/β-catenin signaling in osteoblasts when sclerostin was absent [29] (Table S1). In co-immunoprecipitation (Co-IP) assays under mechanical unloading condition, sclerostin bound LRP4, but it showed no detectable binding to LRP4m (Fig. 2b). To determine the role of sclerostin loop3 and LRP4 interaction in unloading-induced bone formation reduction both *in vitro* and *in vivo*, both *Lrp4m* and LRP4-Pep were utilized in the following structure-function studies under mechanical unloading condition.

Confocal immunofluorescence microscopy showed that *Lrp4m* attenuated the binding of sclerostin to LRP6 in osteoblasts under mechanical unloading condition *in vitro* (Fig. 2c and d). Further, the MC3T3-E1*_Lrp4m* cells cultured in WT MLO-Y4 cell medium under mechanical unloading condition (*Lrp4m*_MUL) had significantly higher Wnt/β-catenin signaling, osteogenic potential and more mineralized nodule formation than the WT MC3T3-E1 cells cultured in WT MLO-Y4 cell medium under mechanical unloading condition (WT_MUL) (Fig. 2e–i). Biotinylated solid-phase binding assay showed that LRP4-Pep attenuated the binding of sclerostin to LRP4 under mechanical unloading condition (Fig. S5). After LRP4-Pep treatment, the WT MC3T3-E1 cells cultured in WT MLO-Y4 cell medium under mechanical unloading condition (WT_MUL + LRP4-Pep) showed significantly higher Wnt/β-catenin signaling, osteogenic potential and more mineralized nodule formation (Fig. 2e–i).

It indicated that the interaction of sclerostin loop3 with LRP4 contributed to the binding of sclerostin to LRP6, and to the decrease of Wnt/β-catenin signaling activity and osteogenic potential in osteoblasts under mechanical unloading condition *in vitro*.

### 2.4 *Lrp4m* (mutation of interaction residues within LRP4 to sclerostin loop3) counteracted unloading-induced bone formation reduction and bone loss in mice

**Fig. 3:**
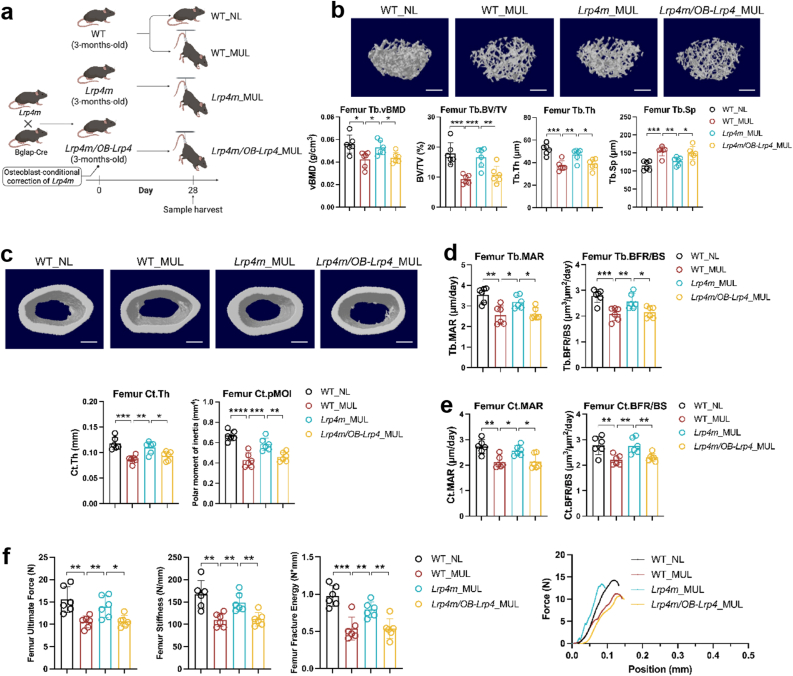
The bone phenotypes of *Lrp4m* mice, *Lrp4m/OB-Lrp4* mice and WT littermate under normal loading or mechanical unloading condition. **(a)** The diagram of experimental design. **(b)** Representative images showing three-dimensional trabecular architecture by micro-CT reconstruction at the distal femur. Scale bars, 200 μm. Bar charts of the structural parameters of Tb.vBMD, Tb.BV/TV, Tb.Th and Tb.Sp from *ex vivo* micro-CT examination at the distal femur (the lower panel). **(c)** Representative images showing three-dimensional cortical architecture by micro-CT reconstruction at the femoral mid-shaft (the upper panel). Scale bars, 200 μm. Bar charts of the structural parameters of Ct.Th and Ct.pMOI from *ex vivo* micro-CT examination at the femoral mid-shaft (the lower panel). **(d)** Analysis of dynamic bone histomorphometric parameters of Tb.MAR and Tb.BFR/BS at the distal femur. **(e)** Analysis of dynamic bone histomorphometric parameters of Ct.MAR and Ct.BFR/BS at the femoral mid-shaft. **(f)** Three-point bending test for femur ultimate force (left), femur stiffness (middle left), and femur fracture energy (middle right). Representative curves showing the mechanical properties of the femoral mid-shaft by three-point bending test (right). Data were expressed as mean ± standard deviation. N = 6 per group. ^ns^ P > *0.05,* ∗P < 0.05, ∗∗P < 0.01, ∗∗∗P < 0.001 and ∗∗∗∗P < 0.0001 for intergroup comparison by unpaired t-test. **NOTE**: NL: normal loading; MUL: mechanical unloading; OB: osteoblast; Tb.vBMD: trabecular volumetric bone mineral density, Tb.BV/TV: trabecular bone volume per total volume; Tb.Th: trabecular thickness; Tb.Sp: trabecular spacing; Ct.Th: cortical thickness; Ct.pMOI: cortical polar moment of inertia; Tb.MAR: trabecular bone mineral apposition rate; Tb.BFR/BS: trabecular bone formation rate; Ct.MAR: cortical bone mineral apposition rate; Ct.BFR/BS: cortical bone formation rate.

For genetic studies *in vivo*, we generated a systemic *Lrp4m* mouse model, as technical constraints precluded an osteoblast-conditional *Lrp4m* mouse model. We then determined bone phenotype of *Lrp4m* mice and their WT littermates, with/without hindlimb unloading for four weeks (Fig. 3a&S6a).

In *micro-CT* analysis, hindlimb unloading led to significantly lower bone mass and worse bone microarchitecture at trabecular bone of distal femur and proximal tibia, as well as at cortical bone of femoral mid-shaft in WT mice (Fig. 3b and c, Fig. S6b). In comparison, *Lrp4m* mice under mechanical unloading condition (*Lrp4m_*MUL) had significantly higher Tb.vBMD, Tb.BV/TV, Tb.Th and lower Tb.Sp at trabecular bone of distal femur, as well as significantly higher Tb.vBMD, Tb.BV/TV, Tb.Th and lower Tb.Sp at trabecular bone of proximal tibia than WT mice under mechanical unloading condition (WT*_*MUL) (Fig. 3b, Fig. S6b). Additionally, *Lrp4m_*MUL mice had significantly higher Ct.Th and Ct.pMOI at cortical bone of femoral mid-shaft than WT*_*MUL mice (Fig. 3c).

In bone histomorphometric analysis, hindlimb unloading caused significantly lower bone formation at trabecular bone of distal femur and proximal tibia, as well as at cortical bone of femoral mid-shaft in WT mice (Fig. 3d and e, Fig. S6c). In comparison, *Lrp4m_*MUL mice had significantly higher Tb.MAR and Tb.BFR/BS at trabecular bone of distal femur, as well as higher Tb.MAR and Tb.BFR/BS at trabecular bone of proximal tibia than WT*_*MUL mice (Fig. 3d, Fig. S6c). Moreover, *Lrp4m_*MUL mice had significantly higher Ct.MAR and Ct.BFR/BS at cortical bone of femoral mid-shaft than WT*_*MUL mice (Fig. 3e).

In the three-point bending test, hindlimb unloading caused significantly weaker bone mechanical properties of femora in WT mice (Fig. 3f). The femur failure force, femur stiffness and femur fracture energy in *Lrp4m_*MUL mice were significantly higher than those in WT*_*MUL mice (Fig. 3f).

It indicated that *Lrp4m* counteracted bone formation reduction and bone loss in mice under mechanical unloading condition.

### 2.5 In *Lrp4m/OB-Lrp4* mice, osteoblast-conditional correction of *Lrp4m* to wild-type *Lrp4* attenuated the counteractive effect of *Lrp4m* on unloading-induced bone loss

To determine whether the counteractive effect of *Lrp4m* on unloading-induced bone formation reduction and bone loss would act on osteoblasts *in vivo*, the *Lrp4m/OB-Lrp4* (OB: osteoblasts) mice with osteoblast-conditional correction of *Lrp4m* to WT *Lrp4* were generated by crossing *Lrp4m* mice with Bglap-Cre mice (Fig. 3a&S6a). The bone phenotype was then determined following hindlimb unloading for four weeks.

In *micro-CT* analysis, *Lrp4m/OB-Lrp4* mice under mechanical unloading condition (*Lrp4m/OB-Lrp4_*MUL) had significantly lower Tb.vBMD, Tb.BV/TV, Tb.Th and higher Tb.Sp at trabecular bone of distal femur (Fig. 3b), as well as significantly lower Tb.vBMD, Tb.BV/TV, Tb.Th and higher Tb.Sp at trabecular bone of proximal tibia than *Lrp4m_*MUL mice (Fig. S6b). Consistently, *Lrp4m/OB-Lrp4_*MUL mice showed significantly lower Ct.Th and Ct.pMOI at cortical bone of femoral mid-shaft than *Lrp4m_*MUL mice (Fig. 3c).

In bone histomorphometric analysis, *Lrp4m/OB-Lrp4_*MUL mice had significantly lower Tb.MAR and Tb.BFR/BS at trabecular bone of distal femur, as well as significantly lower Tb.MAR and Tb.BFR/BS at trabecular bone of proximal tibia than *Lrp4m_*MUL mice (Fig. 3d, Fig. S6c). Moreover, *Lrp4m/OB-Lrp4_*MUL mice had significantly lower Ct.MAR and Ct.BFR/BS at cortical bone of femoral mid-shaft than *Lrp4m_*MUL mice (Fig. 3e).

In the three-point bending test at the femora, *Lrp4m/OB-Lrp4_*MUL mice had significantly lower femur failure force, femur stiffness and femur fracture energy than *Lrp4m_*MUL mice (Fig. 3f).

It indicated that the interaction of sclerostin loop3 with LRP4 in osteoblasts contributed to bone formation reduction and bone loss under mechanical unloading condition *in vivo*.

### 2.6 Osteoblasts-targeted LRP4-Pep counteracted bone formation reduction and bone loss during mechanical unloading in WT mice

For pharmacologic studies *in vivo*, the osteoblasts-targeted LRP4-Pep (5 mg/kg, 10 mg/kg, 20 mg/kg) was subcutaneously administrated into WT mice with hindlimb unloading, once per day for four weeks. Then, bone mass, bone microarchitecture, bone formation and bone mechanical properties were determined (Fig. S7a).

**Fig. 4:**
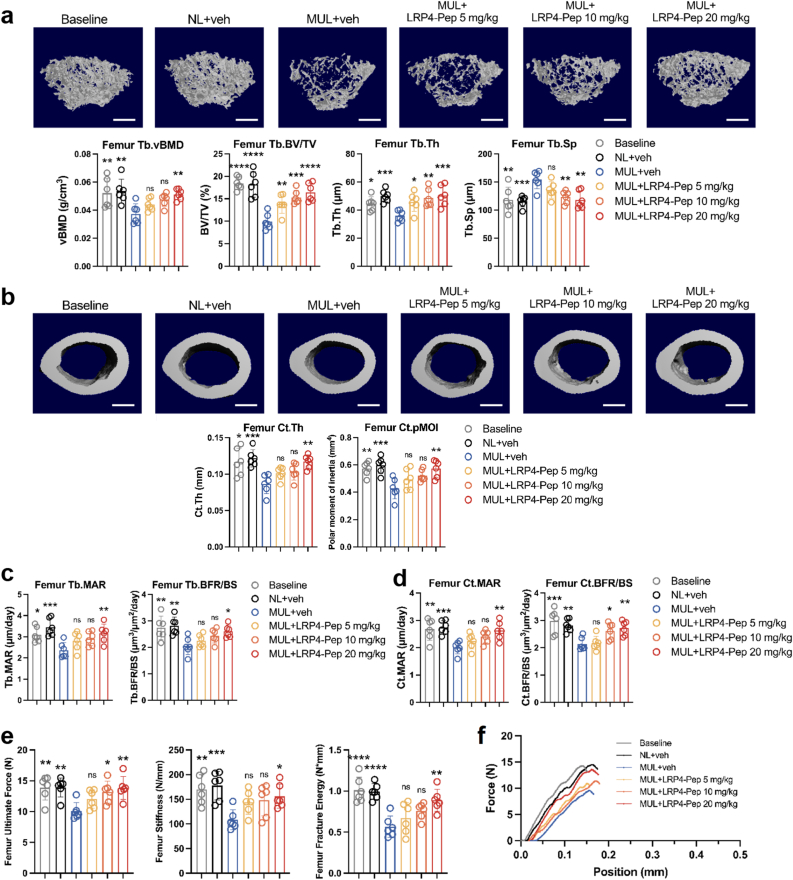
The effects of LRP4-Pep on bone formation in WT mice under MUL condition. **(a)** Representative images showing three-dimensional trabecular bone microarchitecture by micro-CT reconstruction at distal femur. Scale bars, 200 μm (the upper panel). Bar charts of Tb.vBMD, Tb.BV/TV, Tb.Th and Tb.Sp (the lower panel). **(b)** Representative images showing three-dimensional cortical bone microarchitecture by micro-CT reconstruction at femoral mid-shaft (the upper panel). Scale bars, 200 μm. Bar charts of Ct.Th and Ct.pMOI (the lower panel). **(c)** Dynamic bone histomorphometric parameters of Tb.MAR and Tb.BFR/BS at distal femur. **(d)** Dynamic bone histomorphometric parameters of Ct.MAR and Ct.BFR/BS at femoral mid-shaft. **(e)** Three-point bending test for femur ultimate force (left), femur stiffness (middle left), and femur fracture energy (right). **(f)** Representative curves showing mechanical properties of femur. N = 6 per group. ^ns^ P > 0.05, ∗P < 0.05, ∗∗P < 0.01, ∗∗∗P < 0.001, ∗∗∗∗P < 0.0001 for a comparison vs. MUL + veh by one-way ANOVA with Tukey's post-hoc test. **NOTE**: NL: normal loading; MUL: mechanical unloading; Tb.vBMD: trabecular volumetric bone mineral density, Tb.BV/TV: trabecular bone volume per total volume; Tb.Th: trabecular thickness; Tb.Sp: trabecular spacing; Ct. Th: cortical thickness; Ct. pMOI: cortical polar moment of inertia; Tb.MAR: trabecular bone mineral apposition rate; Tb.BFR/BS: trabecular bone formation rate; Ct.MAR: cortical bone mineral apposition rate; Ct.BFR/BS: cortical bone formation rate.

Micro-CT analysis of trabecular bone at distal femur and proximal tibia showed that saline-treated WT mice under mechanical unloading condition (MUL + veh) had significantly lower Tb.vBMD, Tb.BV/TV, Tb.Th, and significantly higher Tb.Sp than saline-treated mice under normal loading condition (NL + veh) (Fig. 4a, Fig. S7d). LRP4-Pep significantly increased Tb.vBMD, Tb.BV/TV, Tb.Th, and decreased Tb.Sp at trabecular bone of both distal femur and proximal tibia in the unloaded mice (MUL + LRP4-Pep), in a dose dependent manner (Fig. 4a, Fig. S7d). Consistently, micro-CT analysis of cortical bone at femoral mid-shaft showed that the MUL + veh mice had significantly lower Ct. Th and Ct.pMOI, compared to the NL + veh mice. LRP4-Pep significantly increased Ct.Th and Ct.pMOI at cortical bone of femoral mid-shaft in the unloaded mice (MUL + LRP4-Pep), in a dose dependent manner (Fig. 4b).

In addition, bone histomorphometric analysis of trabecular bone at distal femur and proximal tibia showed that the MUL + veh mice had significantly lower Tb.MAR and Tb.BFR/BS than the NL + veh mice. LRP4-Pep significantly increased Tb.MAR and Tb.BFR/BS at trabecular bone of distal femur and proximal tibia in the unloaded mice (MUL + LRP4-Pep), in a dose dependent manner (Fig. 4c, Fig. S7b&e). Bone histomorphometric analysis of cortical bone at femoral mid-shaft showed that the MUL + veh mice had significantly lower Ct.MAR and Ct.BFR/BS than the NL + veh mice. LRP4-Pep significantly increased Ct.MAR and Ct.BFR/BS at cortical bone of femoral mid-shaft in the unloaded mice (MUL + LRP4-Pep), in a dose dependent manner (Fig. 4d, Fig. S7c).

Consistent with the data in micro-CT analysis and bone histomorphometric analysis, three-point bending tests at the femora showed significantly lower femur failure force, femur stiffness, and femur fracture energy in MUL + veh mice, compared to those in NL + veh mice. LRP4-Pep significantly enhanced the failure force, stiffness, and fracture energy in the unloaded mice (MUL + LRP4-Pep), in a dose dependent manner (Fig. 4e and f).

Specifically blocking interaction of sclerostin loop3 with LRP4 in osteoblasts by the osteoblasts-targeted LRP4-Pep counteracted bone formation reduction and bone loss under mechanical unloading condition, in a dose dependent manner.

## 3 Discussion

In this study, we notably found that sclerostin loop3-mediated anchoring of sclerostin to LRP4 facilitated its binding to LRP6 in osteoblasts, contributing to bone formation reduction and bone loss under mechanical unloading condition, while the preventive effect of sclerostin against unloading-induced arterial stiffening was independent of sclerostin loop3.

**Regarding bone loss**, we found that either genetic sclerostin loop3-specifc deficiency or pharmacologic sclerostin loop3-specific inhibition by Apc001 counteracted bone formation reduction and bone loss during mechanical unloading in mice. Mechanistically, genetic mutation (*Lrp4m*) study and pharmacologic blockade (LRP4-Pep) study consistently indicated that interaction of sclerostin loop3 with osteoblastic LRP4 facilitated binding of sclerostin to osteoblastic LRP6, contributing to unloading-induced decrease of both Wnt/β-catenin signaling activity and osteogenic potential in osteoblasts *in vitro*, as well as unloading-induced bone formation reduction and bone loss *in vivo*.

Kindlin-2 was reported to mediate mechanotransduction in bone by regulating expression of sclerostin in osteocytes. It suppressed sclerostin expression via downregulating Smad2/3 signaling under mechanical loading, whereas osteocyte-specific Kindlin-2 deficiency elevated sclerostin expression and exacerbated bone loss in both hindlimb unloading and reloading models [35]. While the reported findings established Kindlin-2 as a critical upstream regulator of sclerostin in response to mechanical changes, our study complementarily revealed the downstream mechanism by which sclerostin, once upregulated during unloading, contributed to bone loss.

Under normal loading condition, sclerostin was known to antagonize Wnt/β-catenin signaling pathway in osteoblasts through interaction of sclerostin loop2 with low-density lipoprotein receptor-related proteins 5 and 6 (LRP5/6) [23,36]. In our published studies, we discovered that sclerostin loop3 also participated in the antagonistic effect of sclerostin on bone formation under normal loading condition [25,26]. Consistently here, sclerostin loop3 was found to contribute to bone formation reduction and bone loss under mechanical unloading condition.

Moreover, the key question of how sclerostin loop3 contributes to bone loss under mechanical unloading condition was addressed in this study. It was reported that mechanical unloading led to decrease of Wnt/β-catenin signaling activity in bone of WT mice, accompanied by increased *Sost* expression [14]. During mechanical unloading, we identified LRP4 as the receptor of sclerostin loop3 in osteoblasts. This interaction acted as an anchor that facilitated sclerostin binding to LRP6 in osteoblasts under mechanical unloading condition, contributing to unloading-induced decrease of Wnt/β-catenin signaling activity, bone formation reduction and bone loss. LRP4 was reported to promote sclerostin binding to LRP5/6 for inhibiting bone formation under normal loading condition [30,31,37]. However, whether and how LRP4 facilitates binding of sclerostin to LRP5/6 and contributes to bone loss during mechanical unloading remain to be determined. Here, sclerostin loop3 (rather than sclerostin loop2) was identified as the ligand of LRP4 in osteoblasts that mediated bone loss under mechanical unloading condition.

**Regarding arterial stiffness**, we found that mechanical unloading dramatically enhanced arterial stiffness in mice. Either genetic *Sost* knockout or pharmacologic sclerostin inhibition by the antibody romosozumab further enhanced arterial stiffness, whilst neither sclerostin loop3-specifc deficiency nor sclerostin loop3-specific inhibition by Apc001 produced additional arterial stiffening in mechanical unloaded mice. These findings suggested a loop3-independent preventive role of sclerostin against arterial stiffness in mice.

Bridging the above molecular insights to therapeutic applications, specifically blocking interaction of sclerostin loop3 with LRP4 in osteoblasts, while avoiding inhibition of sclerostin loop2, would offer a precise strategy to counteract bone formation reduction and bone loss without increasing arterial stiffness, under mechanical unloading condition. Future studies utilizing space station-based platforms would be essential to evaluate the long-term efficacy of the developed precise strategy under sustained mechanical unloading condition.

## 4 Experimental design

**Study 1: The effect of sclerostin loop3-specific deficiency/inhibition on unloading-induced bone loss and arterial stiffness.** To determine whether sclerostin loop3 could contribute to unloading-induced bone formation reduction and bone loss, as well as to unloading-induced increase of arterial stiffness *in vivo*, the 3-month-old male wild-type (C57BL/6 J, WT) mice, *Sost*^*−/−*^ mice and *Sost*^*loop3−/*^^*−*^ mice were randomized into the following groups (n = 6 per group): (1) WT_NL, (2) WT_MUL, (3) *Sost*^*−/−*^_MUL, (4) *Sost*^*loop3−/*^^*−*^_MUL and (5) WT_MUL + Apc001. For group (1), the WT mice were reared under normal loading (NL) condition for 4 weeks as control. For groups (2), (3) and (4), the WT mice, *Sost*^*−/−*^ mice and *Sost*^*loop3−/*^^*−*^ mice were subjected to hindlimb unloading by tail suspension (mechanical unloading: MUL) for four weeks, respectively. For group (5), the WT mice were subjected to hindlimb unloading and were subcutaneously administrated with Apc001 (50 mg/kg) once per week for four weeks. Before euthanasia, all mice were injected intraperitoneally with calcein green (20 mg/kg) at 10 and 2 days, respectively. After sacrifice, the heart, carotid artery and aorta were carefully dissected, exposed and marked. Carotid artery stiffness was determined by doppler ultrasound analysis [28]. The left femur and left tibia were collected for examining bone mass and bone microarchitecture by micro-CT analysis, followed by bone histomorphometric analysis. The right femora were wrapped with PBS-soaked gauze and stored at −80 °C for further examination of the bone mechanical properties by three-point bending test [25,26,[38], [39], [40], [41], [42]].

**Study 2: The effect of osteoblastic *Lrp4* knockout on unloading-induced bone loss.** To determine the role of osteoblastic *Lrp4* in unloading-induced bone formation reduction and bone loss *in vivo*, we established the osteoblast-specific *Lrp4* knockout (*OB*.*Lrp4*^*−/−*^) mouse model. The 3-month-old male WT mice and *OB*. *Lrp4*^*−/−*^ mice were randomized into the following groups (n = 6 per group): (1) WT_NL, (2) WT_MUL, and (3) *OB*.*Lrp4*^*−/−*^_MUL. For group (1), the WT mice were reared under normal loading condition for 4 weeks as control. For groups (2) and (3), WT mice and *OB*. *Lrp4*^*−/−*^ mice were subjected to hindlimb unloading by tail suspension for four weeks, respectively. Before euthanasia, all mice were injected intraperitoneally with calcein green (20 mg/kg) at 10 and 2 days, respectively. After sacrifice, the left femur and left tibia were collected for examining bone mass and bone microarchitecture by micro-CT analysis, followed by bone histomorphometric analysis. The right femora were wrapped with PBS-soaked gauze and stored at −80 °C for further examination of the bone mechanical properties by three-point bending test [25,26,38].

**Study 3: The role of sclerostin loop3-LRP4 interaction in unloading-induced decreased Wnt/β-catenin signaling activity and osteogenic potential in osteoblasts *in vitro*.** We used the conditional medium of osteocytes (MLO-Y4 cells) under normal loading or mechanical unloading condition to culture osteoblasts (MC3T3-E1 cells) under normal loading or mechanical unloading condition. The *in vitro* mechanical unloading studies were conducted utilizing the desktop Random Positioning Machine (RPM), a ground-based rotational microgravity simulator [43].

We identified the LRP4 residues (Y200, G201, L205, D206, I207, Y208, H209, C210) that interacted with sclerostin loop3. Then, *Lrp4* mutation tool (*Lrp4m*, encoding LRP4m-Y200A/G201A/Y208A/H209A/C210A) and osteoblasts-targeted LRP4 blocking peptide tool (LRP4-Pep, residues P190-S226, an LRP4 LA5 fragment conjugated to the bone-formation-surface-targeting oligopeptide (DSS)_6_) [33,34] were engineered to specifically block the interaction of sclerostin loop3 with LRP4, without altering Wnt/β-catenin signaling in osteoblasts when sclerostin was absent [29]. In this study, the blockade effects of LRP4-Pep on binding between sclerostin and LRP4 under mechanical unloading condition were determined by biotinylated solid-phase binding assay. The binding between sclerostin and LRP4(m) in osteoblasts (MC3T3-E1) under mechanical unloading condition were determined by co-immunoprecipitation (Co-IP) analysis *in vitro*. Then, the influence of *Lrp4m* in the binding between sclerostin and LRP6 in osteoblasts (MC3T3-E1) under mechanical unloading condition was detected by confocal immunofluorescence microscopy *in vitro*.

To determine the effect of *Lrp4m* and LRP4-Pep on Wnt/β-catenin signaling and osteogenic potential in osteoblasts under mechanical unloading condition, MC3T3-E1 cells were divided into 5 groups (n = 3 per group), including (1) WT_NL, (2) WT_MUL, (3) *Sost*^*−/−*^_MUL, (4) *Lrp4m*_MUL and (5) WT_MUL + LPR4-Pep. In group (1), the WT MC3T3-E1 cells were cultured in conditional medium of normal loaded WT MLO-Y4 cells under normal loading (NL) condition. In groups (2) and (4), the WT MC3T3-E1 cells or MC3T3-E1_*Lrp4m* cells were cultured in conditional medium of mechanical unloaded WT MLO-Y4 cells under mechanical unloading (MUL) condition. In group (3), the MC3T3-E1*_Sost*^*−/−*^ cells were cultured in conditional medium of mechanical unloaded MLO-Y4_*Sost*^*−/−*^ cells under mechanical unloading condition. In group (5), the WT MC3T3-E1 cells were cultured in conditional medium of mechanical unloaded WT MLO-Y4 cells, with treatment of LRP4-Pep, under mechanical unloading condition. After 48 h, all groups of MC3T3-E1 cells were collected for determination of Wnt/β-catenin signaling by TOP-Wnt-induced luciferase reporter assay. After 3 days, all groups of MC3T3-E1 cells were collected for determination of mRNA expression levels of β-catenin by RT-qPCR. After 7 days, all groups of MC3T3-E1 cells were collected for determination of mRNA or protein expression levels of ALP by RT-qPCR or ALP staining, respectively. After 21 days, all groups of MC3T3-E1 cells were collected for determination of mRNA or protein expression levels of OCN by RT-qPCR or Alizarin Red S solution staining, respectively [25].

**Study 4: Genetic determination of the counteractive effect of osteoblastic *Lrp4m* on bone formation reduction and bone loss during mechanical unloading *in vivo*.** For genetic studies *in vivo*, technical constraints precluded the development of the osteoblast-conditional *Lrp4m* mouse model. Thus, we developed systematic *Lrp4m* mouse model (C57BL/6 J). Then, we crossbred *Lrp4m* mice with Bglap-Cre mice to develop the *Lrp4m/OB-Lrp4* mouse model (OB: osteoblasts) in which only osteoblasts had no mutations in *Lrp4*. The 3-month-old male *Lrp4m* mice, *Lrp4m/OB-Lrp4* mice and WT littermates (n = 6 per group) were subjected to hindlimb unloading with tail suspension for four weeks (mechanical unloading). Meanwhile, 3-month-old male WT littermates (n = 6) were reared under normal loading condition for 4 weeks as control. Before euthanasia, all mice were injected intraperitoneally with calcein green (20 mg/kg) at 10 and 2 days, respectively. After sacrifice, the left femur and left tibia were collected for examining bone mass and bone microarchitecture by micro-CT analysis, followed by bone histomorphometric analysis. The right femora were wrapped with PBS-soaked gauze and stored at −80 °C for further examination of the bone mechanical properties by three-point bending test [25,26,38].

**Study 5: Pharmacologic determination of the counteractive effect of osteoblasts-targeted LRP4-Pep on bone formation reduction and bone loss during mechanical unloading *in vivo*.** For pharmacologic studies *in vivo*, the developed osteoblasts-targeted LRP4-Pep (end-protected by changing the first and last three amino acids from L-type to D-type [44,45], conjugated to our developed bone-formation-surface-targeting oligopeptide (DSS)_6_ [33,34]) was subcutaneously administrated in the following studies. The 3-month-old male WT mice (C57BL/6 J) were randomized into the following groups (n = 6 per group): (1) Baseline, (2) NL + veh, (3) MUL + veh, (4) MUL + LRP4-Pep 5 mg/kg, (5) MUL + LRP4-Pep 10 mg/kg, (6) MUL + LRP4-Pep 20 mg/kg. For group (1), the WT mice were sacrificed at 3-month-old as baseline. For group (2), the WT mice were reared under normal loading condition for 4 weeks as control. For groups (3)-(6), the WT mice were subjected to hindlimb unloading with tail suspension and subcutaneously injected with saline (veh) or osteoblasts-targeted LRP4-Pep at dosage of 5 mg/kg, 10 mg/kg, 20 mg/kg, respectively, once per day for four weeks. Before euthanasia, all mice were injected intraperitoneally with calcein green (20 mg/kg) at 10 and 2 days, respectively. After sacrifice, the left femur and left tibia were collected for examining bone mass and bone microarchitecture by micro-CT analysis, followed by bone histomorphometric analysis. The right femora were wrapped with PBS-soaked gauze and stored at −80 °C for further examination of the bone mechanical properties by three-point bending test [25,26,38].

## 5 Evaluation protocols

**Mice and genotyping.** The *sost*^*−/−*^ mice, *Sost*^*loop3−/*^^*−*^ mice, *Lrp4*^*flox/flox*^ mice and *Lrp4m* mice (encoding LRP4-Y200A, G201A, Y208A, H209A, C210A), the and the Bglap-Cre mice were constructed in collaboration with GemPharmatech Co., Ltd, China. Mouse genotypes were determined by PCR on tail genomic DNA. The *sost*^*−/−*^ mice was genotyped using the following primers: 5′-AGTGATATGGTGAGGCTGGATGC-3′ and 5′-GAACCTCAGTGATGGCTTAGTGG-3′ for *sost*^*−/−*^ allele (555 bp), 5′-ACACACAATGTCTCGCCACTGT-3′ and 5′-CAGCTAACTGAAGAGACAGGGATAG-3′ for WT allele (378 bp). The *Sost*^*loop3−/*^^*−*^ mice was genotyped using the following primers: 5′-GACTACAAAGACCATGACGGTGATT-3′ and 5′-TACACTGAAAACGAATCGGATCGC-3′ for *Sost*^*loop3−/*^^*−*^ allele (121 bp), and 5′-GGTGCCTCCTTCCTATAATCCATA-3′ and 5′-GAGGCCACCAGACGCACCTT-3′ for WT allele (398 bp). The *Lrp4*^*flox/flox*^ mice were genotyped using the following primers: 5′- AACACAGGAGGCAGCAAGAT-3′ and 5′- GATCCTGGCAAGAAAGGGGT-3′, as well as 5′-CCACCTAAGGTCCTGGTTCG-3′ and 5′- GATCGATCCTACCCCTTGCG-3′ for *Lrp4*^*flox/flox*^ allele (270 bp and 691 bp, respectively). The *Lrp4m* mice were genotyped using the following primers: 5′-TTGGGTGGACAGGCATCAATG-3′ and 5′-CCTCCCTGAATAACTTCGTATAATGTATGC-3′ for the 5′ arm of *Lrp4* allele (1567 bp), 5′-GATCCCCATCAAGCTGATAACATACG-3′ and 5′-GACACTCACGGCAGCTTTCCTC-3′ for the 3′ arm of *Lrp4* allele (1929 bp), and 5′-GGGAACGAAGCTACAACCTGGAC-3′ and 5′-CGACAGTCCTGCTCATCAGAGTC-3′ for WT allele (835 bp). The Bglap-Cre mice was genotyped using the following primers: 5ʹ-GGGCAGTCTGGTACTTCCAAGCT-3ʹ and 5ʹ-ATTGTGGTGCAGCCAAGCTGCTA-3ʹ for *Cre* allele (304 bp), 5ʹ-AGTCTTTCCCTTGCCTCTGCT-3ʹ and 5′- GGGTCTTCCACCTTTCTTCAG -3ʹ for WT allele (825 bp). The obtained heterozygous *Lrp4*^*flox/flox*^ mice and *Lrp4m* mice were used for subsequent crossbreeding with Bglap-Cre mice to generate *OB. Lrp4*^*−/−*^ mice and *Lrp4m/OB-Lrp4* mice, respectively [25].

***Micro-CT* analysis.** *Micro-CT* analysis was used for analysis of the trabecular/cortical bone mass and bone architecture. The collected left femur and tibia were fixed with 4% PFA and scanned using a *Micro-CT* (version 6.5, vivaCT40, SCANCO Medical AG). Briefly, a total of 424 slices with a voxel size of 10 μm were scanned at the region of the proximal tibia/distal femur beginning at the growth plate and extending distally along the tibial/femoral diaphysis. Regions of interest (ROIs) were defined using Scanco evaluation software. Images of femurs and tibias were reconstructed and segmented (200 m s integration time, 0.8 sigma, 1 support, 260 thresholds). For the proximal tibia/distal femur 100 sequential slices beginning at 0.1 mm from the most proximal aspect of the growth plate, in which both condyles were no longer visible, were selected for analysis. The trabeculae were analyzed by manual contouring excluding the cortical bone. Trabecular bone parameters, including trabecular volume per total volume (Tb.BV/TV), trabecular volumetric bone mineral density (Tb.vBMD), trabecular thickness (Tb.Th) and trabecular spacing (Tb.Sp) were calculated. For the femoral mid-shaft, 100 slices were measured at the exact center and at the distal 50% of femur length using the automated thresholding algorithm. Trabeculae in contact with cortical bone were manually removed from the ROI. Cortical bone parameters, including cortical thickness (Ct.Th) and cortical polar moment of inertia (Ct.pMOI) were calculated [25,26].

**Bone histomorphometric analysis.** Calcein (20 mg/kg, Sigma, C0875) was intraperitoneally administered 10 and 2 days before euthanasia. The collected undecalcified right femur samples were dehydrated in increasing concentrations of sucrose (10%, 20%, 30% in PBS) for 24 h at each concentration and embedded in an optimal cutting temperature O.C.T. compound (Sakura Finetek). Frontal sections (thickness: 7 μm) of trabecular bone were obtained from the femoral metaphysis by longitudinal cryosection with CryoStar NX50 (Thermo Fisher Scientific). Cross sections (thickness: 7 μm) of cortical bone were obtained from the femoral mid-shaft. Fluorescence micrographs of the bone sections with calcein green labels were captured by a Q500MC fluorescence microscope (Leica). Dynamic parameters including the mineral apposition rate (MAR) and bone formation rate (BFR/BS) were analyzed and calculated according to ASBMR standardized nomenclature for bone histomorphometry [26,46,47].

**Bone mechanical testing.** The right femora were collected, wrapped in PBS-moistened gauze and stored at −80 °C after sacrifice. Prior to three-point bending test, these frozen bone samples were naturally thawed to room temperature. Three-point bending test was performed using a universal testing machine (H25KS Series, Hounsfield Test Equipment Ltd.) equipped with a 2.5 kN load cell. Femurs were loaded in the anterior-posterior direction with the span length set as 17 mm. Load was applied with a constant displacement rate of 1 mm/min at the femur mid-shaft. After failure, the load vs. displacement curves were recorded, and the ultimate force (N), stiffness and fracture energy (J) were calculated for statistical analysis [25,26,38].

**Arterial stiffness testing.** Arterial stiffness testing was performed using a Vevo 3100 L T high-resolution ultrasound system (FUJIFILM VisualSonics, Toronto, Canada), equipped with a MX550D transducer (transmit frequency 32 MHz, frequency range 25-55 MHz). Two-dimensional and Doppler ultrasonography were used to visualize and define sampling sites on the mouse common carotid artery and aortic arch, and to obtain blood flow velocity spectra at these locations. The carotid sampling point was set 0.5 cm proximal to the common carotid bifurcation. After marking the site on the body surface, the animal was euthanized. The heart, common carotid artery, and aorta were carefully dissected and exposed. A moistened thread was laid along the vascular course to measure the actual path length between the marked carotid point and the aortic arch. This was defined as the heart-carotid distance. On the Doppler velocity spectra, using the time scale, the interval between the foot (onset) of the waveform and the peak of the ECG R wave was measured at the aortic site (TR-aortic) and at the carotid site (TR-carotid). The heart-carotid pulse wave transit time was calculated as TR-carotid minus TR-aortic, and the pulse wave velocity (PWV) for this arterial segment was derived by dividing the heart-carotid path length by the transit time [28].

**Pull-down assay.** To conduct pull-down assay under mechanical unloading condition *in vitro*, the MC3T3-E1 cell lysates were collected using RIPA Lysis and Extraction Buffer (Thermo Scientific, 89,901) supplemented with a Protease Inhibitor Cocktail (Cell Signaling Technology, 5871). The lysate was then centrifuged at 4000 rpm for 30 min at 4 °C and replaced with binding buffer (PBS buffer, 20 mM imidazole, pH 8.0). Following three rounds of pre-equilibration with binding buffer, 20 μL of Ni-NTA Magnetic Agarose Beads were incubated with 6 μg of His-tagged sclerostin (His-SOST) or 6 μg of His-tagged sclerostin loop3 (His-SOST loop3) in binding buffer for 2 h at 4 °C with gentle rotation. The beads carrying His-SOST or His-SOST loop3 were then incubated with 500 μL cell lysate supernatant at 4 °C overnight under mechanical unloading condition utilizing the desktop Random Positioning Machine (RPM, Airbus Defense and Space, Netherlands. Software: RPMSW) [43]. To eliminate the effects of non-specific binding between beads and cell lysate on targeted interaction identification, 20 μL beads and 500 μL cell lysate were incubated under the same unloading conditions as control. After incubation, the beads were washed 3 times with 500 μL washing buffer (PBS buffer, 20 mM imidazole, 0.005% Tween 20, pH 8.0), and finally eluted with elution buffer (PBS buffer, 250 mM imidazole, 0.005% Tween 20, pH 8.0) [48]. Then, western blots were conducted with anti-His antibody (Abcam, ab245114, diluted at 1:1000) and anti-LRP4 antibody (Invitrogen, PA5-68,218, diluted at 1:1000) [49].

**Biotinylated solid-phase binding assay.** A 96-well plate was coated with 200 ng of LRP4 and negative controls per well at 4 °C overnight, followed by the incubation of blocking buffer (PBS, 1% BSA, 1% Tween 20) at 25 °C for 1 h. After washing with washing buffer (PBS, 0.1% BSA, 0.1% Tween 20), biotinylated sclerostin (100 μL/well) with/without pre-incubation of Apc001/LRP4-Pep was added and incubated at 25 °C for 2 h under mechanical unloading condition utilizing the desktop Random Positioning Machine (RPM, Airbus Defense and Space, Netherlands. Software: RPMSW) [43]. Non-specifically bound sclerostin was removed by four 5-min washes with washing buffer. Then, 100 μL streptavidin-HRP was added and incubated for 30 min, followed by four 5-min washes with washing buffer. 50 μL 3,3′,5,5′-tetramethylbenzidine (TMB) was added and incubated for 20 min. The reaction was stopped with 50 μL H_2_SO_4_ (2 mol/L), and absorbance at 450 nm was measured using a Molecular Device i3x microplate reader. Data were analyzed with Origin 8.0 [50,51].

**Enzyme-linked immunosorbent assay (ELISA).** The protein levels of sclerostin were detected using the sclerostin ELISA kits (R&D, DSST00; Cloud-Clone, SEC864Mu) in triplicate following the manufacturer's instructions [25].

**Co-immunoprecipitation (Co-IP) analysis.** MC3T3-E1 cells were overexpressed with His-tagged sclerostin (His-SOST) and Flag-tagged WT LRP4 (Flag-LRP4) or overexpressed with His-tagged sclerostin (His-SOST) and Flag-tagged LRP4m (Flag-LRP4m), respectively (Lipomaster 3000 Transfection Reagent, Vazyme Biotech Co. Ltd, TL301). After culturing cells under mechanical unloading condition utilizing the desktop Random Positioning Machine (RPM, Airbus Defense and Space, Netherlands. Software: RPMSW) [43] for 48 h, cell lysates were harvested and incubated with 20 μL Ni-NTA Magnetic Agarose Beads, in binding buffer (PBS buffer, 20 mM imidazole, pH 8.0) for 2 h at 4 °C with gentle rotation. Then, the beads were washed 3 times with 500 μL wash buffer (PBS buffer, 20 mM imidazole, 0.005% Tween 20, pH 8.0), and finally eluted with elution buffer (PBS buffer, 250 mM imidazole, 0.005% Tween 20, pH 8.0) [48]. Western blots were conducted with anti-His antibody (Abcam, ab245114, diluted at 1:1000), and anti-Flag antibody (Abcam, ab125243, diluted at 1:2000) [49].

**Confocal immunofluorescence microscopy.** The WT MC3T3-E1 cells or MC3T3-E1_*Lrp4m* cells were cultured with a density of 1 × 10^5^ cells per well in α-MEM medium supplemented with 10% FBS and 1% P/S at the confocal dish (Thermo, 150,680) under mechanical unloading condition for 12 h. After being fixed with 4% PFA, the cells were incubated with anti-LRP6 antibody (Creative Biolabs, CBYCL-470, 1:100) and anti-sclerostin antibody (Thermo, PA5-113,315, 1:100) overnight at 4 °C, followed by incubation with goat anti-mouse IgG H&L (Alexa Fluor 594, 5 μg/mL) and goat anti-rabbit IgG H&L (Alexa Fluor 488, 5 μg/mL), respectively, for 2 h at 37 °C. Hoechst was used to stain the cell nuclei. The confocal microscope (Leica, STELLARIS STED) was utilized to obtain fluorescent images. ImageJ software was used to measure fluorescence integrated density [52].

**TOP-Wnt-induced luciferase reporter assay.** The WT MC3T3-E1 cells or MC3T3-E1_*Lrp4m* cells were co-transfected with the corresponding reporter plasmids including TOPFlash, sv40 and Wnt3a, using Lipomaster 3000 transfection reagent (Vazyme, TL301). After 6 h, the culture medium was changed to the conditional medium of mechanical unloaded MLO-Y4 cells, with or without LRP4-Pep. The MC3T3-E1 cells were cultured under mechanical unloading condition utilizing the desktop Random Positioning Machine (RPM, Airbus Defense and Space, Netherlands. Software: RPMSW) [43] for 48 h. Then, the cells were lysed with 100 μL/well passive lysis buffer. Luciferase assays were performed using the Dual-Luciferase Reporter Assay System (Promega, E1980) according to the manufacturer's protocol [53,54].

**Real-time quantitative PCR (RT-qPCR).** Total RNA from cultured cells was isolated by homogenization using TRIzol (Invitrogen, 15,596,026) and reverse transcribed into cDNA using a HiScript III RT SuperMix kit (Vazyme, R323). RT-qPCR was performed on the QuantStudio 7 PRO System (Applied Biosystems). Bone formation biomarkers’ expression was determined using primers including those for Sost (F: AGCCTTCAGGAATGATGCCAC, R: CTTTGGCGTCATAGGGATGGT), GAPDH (F: AGGTCGGTGTGAACGGATTTG, R: GGGGTCGTTGATGGCAAC), β-catenin (F: GCCACAGGATTACAAGAAGCGG, R: GGCACCAATGTCCAGTCCAAG), ALP (F: AACCCAGACACAAGCATTCC, R: GCCTTTGAGGTTTTGGGTCA), and Bglap (OCN, F: CAGCCACCGAGACACCAT, R: CCAGCAGAGCGACACCCTA). Relative mRNA expression of genes was determined using the 2^−ΔΔCt^ method with GAPDH as the endogenous normalizer [55].

**Alkaline phosphatase (ALP) staining.** After 7 days of osteogenic differentiation under mechanical unloading condition (RPM, Airbus Defense and Space, Netherlands. Software: RPMSW) [43], ALP staining was performed with BCIP/NBT Alkaline Phosphatase Kit (Beyotime) with p-nitrophenyl phosphate (pNPP) as the chromogenic substrate, according to manufacturer's instructions [[56], [57], [58]].

**Alizarin red staining.** After 21 days of osteogenic differentiation under mechanical unloading condition (RPM, Airbus Defense and Space, Netherlands. Software: RPMSW) [43], Alizarin Red S solution (40 mM, pH 4.2, Cyagen, ALIR-10001) was used to evaluate extracellular matrix mineralization in mature osteoblasts [57]. Cells were then washed three times with distilled water and examined for the presence of calcium deposits. Then, the calcium-bound dye was removed with pH neutral 10% cetylpyridinium chloride in 10 mM sodium phosphate buffer. Mineralization was quantified by a SpectraMax i3x Multi-Mode Microplate Reader (Molecular devices) at 550 nm wavelength [59,60].

**Statistical analysis.** Comparisons between groups were carried out using the unpaired Student's t-test or One-Way ANOVA with Tukey's post-hoc test. Error bars indicated the mean ± standard deviation. All statistical data were analyzed by GraphPad Prism (version 8; GraphPad Software), and *P* < 0.05 was considered statistically significant.

## Author contributions

G. Zhang, L. Wang, B. Zhang, and S. Zhang supervised the project. L. Wang, N. Zhang, H. Jiang, X. Tao, Y. Ma, Z. Hu and C. Zhong jointly performed the major research, wrote and edited the manuscript. S. Yu, H. Zhang, Z. Chen, H. Luo and Y. Zhang synthesized the Apc001 and osteoblast-targeted LRP4-Pep. X. Yang, S. Ding, and M. Sun contributed to genotyping and micro-CT analysis. L. Qin, A. Lu, T. Zhang, P. Ferdinandy, S.Q. Zhang, J. Liu, Y. Yu, and F. Liu provided their professional expertise. All authors revised the content and approved the submitted version.

We acknowledge the Language Center at Hong Kong Baptist University for language-proofreading support. The schematic diagram was created using BioRender.com.

## Ethics approval

All animal procedures were approved by the Department of Health (ethic approval number: REC/24-25/0061), the Government of the Hong Kong Special Administrative Region, as well as by the Research Ethics Committee of Hong Kong Baptist University.

## Declaration of generative AI in scientific writing

No generative artificial intelligence (AI) or AI-assisted technologies were used in the preparation of this manuscript.

## Funding statement

This study was supported by the Shenzhen-Hong Kong-Macau Science and Technology Plan Project (Category C), China (Grant No. SGDX20230821095359002), Hong Kong General Research Fund from the Research Grants Council of the Hong Kong Special Administrative Region, China (Project No. 12100725, Project No. 12102223, Project No. 12102524, Project No. 12100921), Theme-based Research Scheme from the Research Grants Council of the Hong Kong Special Administrative Region, China (Project No. T12-201/20-R), Young Scientists Fund of the National Natural Science Foundation of China, China (Grant No. 82300988, Grant No. 22507110), Guangdong Provincial Natural Science Foundation - General Programme, China (Project No. 2026A1515010818), Inter-institutional Collaborative Research Scheme from Hong Kong Baptist University, the Hong Kong Special Administrative Region, China (Project No. RC-ICRS/19-20/01), University-Industry Collaboration Programme from Innovation and Technology Commissions of the Hong Kong Special Administrative Region, China (Project No. UIM/298), and University-Industry Collaboration Programme from Innovation and Technology Commissions of the Hong Kong Special Administrative Region, China (Project No. UIM/328).

## Conflict of interests

The authors declare no conflict of interests.

## Data Availability

The data used to support the findings of this study are available from the corresponding author upon request.

## References

[1] Rolvien T., Amling M. (2022). Disuse osteoporosis: clinical and mechanistic insights. Calcif Tissue Int.

[2] Grimm D., Grosse J., Wehland M., Mann V., Reseland J.E., Sundaresan A. (2016). The impact of microgravity on bone in humans. Bone.

[3] Li C.Y., Price C., Delisser K., Nasser P., Laudier D., Clement M. (2005). Long-term disuse osteoporosis seems less sensitive to bisphosphonate treatment than other osteoporosis. J Bone Miner Res.

[4] Sato Y., Asoh T., Kaji M., Oizumi K. (2000). Beneficial effect of intermittent cyclical etidronate therapy in hemiplegic patients following an acute stroke. J Bone Miner Res.

[5] Pajevic P.D., Spatz J.M., Garr J., Adamson C., Misener L. (2013). Osteocyte biology and space flight. Curr Biotechnol.

[6] Uda Y., Azab E., Sun N., Shi C., Pajevic P.D. (2017). Osteocyte mechanobiology. Curr Osteoporos Rep.

[7] Spatz J.M., Wein M.N., Gooi J.H., Qu Y., Garr J.L., Liu S. (2015). The wnt inhibitor sclerostin is Up-regulated by mechanical unloading in osteocytes in vitro. J Biol Chem.

[8] Macaulay T.R., Siamwala J.H., Hargens A.R., Macias B.R. (2017). Thirty days of spaceflight does not alter murine calvariae structure despite increased sost expression. BoneKEy Rep.

[9] Smith S.M., Heer M., Shackelford L.C., Sibonga J.D., Spatz J., Pietrzyk R.A. (2015). Bone metabolism and renal stone risk during international space station missions. Bone.

[10] Belavý D.L., Baecker N., Armbrecht G., Beller G., Buehlmeier J., Frings-Meuthen P. (2016). Serum sclerostin and DKK1 in relation to exercise against bone loss in experimental bed rest. J Bone Miner Metabol.

[11] Spatz J.M., Fields E.E., Yu E.W., Divieti Pajevic P., Bouxsein M.L., Sibonga J.D. (2012). Serum sclerostin increases in healthy adult men during bed rest. J Clin Endocrinol Metab.

[12] Spatz J.M., Ellman R., Cloutier A.M., Louis L., van Vliet M., Suva L.J. (2013). Sclerostin antibody inhibits skeletal deterioration due to reduced mechanical loading. J Bone Miner Res.

[13] Osumi R., Wang Z., Ishihara Y., Odagaki N., Iimura T., Kamioka H. (2021). Changes in the intra- and peri-cellular sclerostin distribution in lacuno-canalicular system induced by mechanical unloading. J Bone Miner Metabol.

[14] Lin C., Jiang X., Dai Z., Guo X., Weng T., Wang J. (2009). Sclerostin mediates bone response to mechanical unloading through antagonizing wnt/Beta-Catenin signaling. J Bone Miner Res.

[15] Qin W., Zhao W., Li X., Peng Y., Harlow L.M., Li J. (2016). Mice with sclerostin gene deletion are resistant to the severe sublesional bone loss induced by spinal cord injury. Osteoporos Int.

[16] Tian X., Jee W.S., Li X., Paszty C., Ke H.Z. (2011). Sclerostin antibody increases bone mass by stimulating bone formation and inhibiting bone resorption in a hindlimb-immobilization rat model. Bone.

[17] Lewiecki E.M., Blicharski T., Goemaere S., Lippuner K., Meisner P.D., Miller P.D. (2018). A phase III randomized placebo-controlled trial to evaluate efficacy and safety of romosozumab in men with osteoporosis. J Clin Endocrinol Metab.

[18] Saag K.G., Petersen J., Brandi M.L., Karaplis A.C., Lorentzon M., Thomas T. (2017). Romosozumab or alendronate for fracture prevention in women with osteoporosis. N Engl J Med.

[19] Wieser M., Gisler S., Sarabadani A., Ruest R.M., Buetler L., Vallery H. (2014). Cardiovascular control and stabilization via inclination and mobilization during bed rest. Med Biol Eng Comput.

[20] Palombo C., Morizzo C., Baluci M., Lucini D., Ricci S. (2015). Large artery remodeling and dynamics following simulated microgravity by prolonged head-down tilt bed rest in humans. BioMed Res Int.

[21] Hughson R.L., Robertson A.D., Arbeille P., Shoemaker J.K., Rush J.W., Fraser K.S. (2016). Increased postflight carotid artery stiffness and inflight insulin resistance resulting from 6-mo spaceflight in Male and female astronauts. Am J Physiol Heart Circ Physiol.

[22] Luyao Wang T.X., Zhong Chuanxin, Xv Liqun, Tao Xiaohui, Hu Zebing, Zhang Shu (2023).

[23] Holdsworth G., Slocombe P., Doyle C., Sweeney B., Veverka V., Le Riche K. (2012). Characterization of the interaction of sclerostin with the low density lipoprotein receptor-related protein (LRP) family of wnt co-receptors. J Biol Chem.

[24] Bourhis E., Wang W., Tam C., Hwang J., Zhang Y., Spittler D. (2011). Wnt antagonists bind through a short peptide to the first beta-propeller domain of LRP5/6. Structure.

[25] Yu Y., Wang L., Ni S., Li D., Liu J., Chu H.Y. (2022). Targeting loop3 of sclerostin preserves its cardiovascular protective action and promotes bone formation. Nat Commun.

[26] Wang L., Yu Y., Ni S., Li D., Liu J., Xie D. (2022). Therapeutic aptamer targeting sclerostin loop3 for promoting bone formation without increasing cardiovascular risk in osteogenesis imperfecta mice. Theranostics.

[27] Zhang J., Wang X., Fu Z., Xing C., Wang Z., Yang H. (2024). Long-term simulated microgravity fosters carotid aging-like changes via Piezo1. Cardiovasc Res.

[28] Di Lascio N., Kusmic C., Stea F., Faita F. (2017). Ultrasound-based pulse wave velocity evaluation in mice. J Vis Exp.

[29] Luyao Wang X.T., Jiang Hewen, Zhang Ning, Li Dijie, Liu Jin, Yu Yuanyuan (2023). Sclerostin Loop3-LRP4 interaction required by sclerostin for antagonizing wnt signaling and osteogenic potential in osteoblasts. J Bone Miner Res.

[30] Xiong L., Jung J.U., Wu H., Xia W.F., Pan J.X., Shen C. (2015). Lrp4 in osteoblasts suppresses bone formation and promotes osteoclastogenesis and bone resorption. Proc Natl Acad Sci USA.

[31] Chang M.K., Kramer I., Huber T., Kinzel B., Guth-Gundel S., Leupin O. (2014). Disruption of Lrp4 function by genetic deletion or pharmacological blockade increases bone mass and serum sclerostin levels. Proc Natl Acad Sci USA.

[32] Jiang H., Tao X., Yu S., Zhang Y., Ma Y., Li N. (2026). Adipocytic sclerostin loop3-LRP4 interaction required by sclerostin to impair whole-body lipid and glucose metabolism. Nat Commun.

[33] Zhang G., Guo B., Wu H., Tang T., Zhang B.T., Zheng L. (2012). A delivery system targeting bone formation surfaces to facilitate RNAi-based anabolic therapy. Nat Med.

[34] Zhuo Z., Wan Y., Guan D., Ni S., Wang L., Zhang Z. (2020). A loop-based and AGO-incorporated virtual screening model targeting AGO-mediated miRNA-mRNA interactions for drug discovery to rescue bone phenotype in genetically modified mice. Adv Sci.

[35] Qin L., Fu X., Ma J., Lin M., Zhang P., Wang Y. (2021). Kindlin-2 mediates mechanotransduction in bone by regulating expression of sclerostin in osteocytes. Commun Biol.

[36] Holdsworth G., Roberts S.J., Ke H.Z. (2019). Novel actions of sclerostin on bone. J Mol Endocrinol.

[37] Svetlana K., Biplab C., Chen V.A.D., Niv P., Noam L. (2022). Competitive blocking of LRP4–sclerostin binding interface strongly promotes bone anabolic functions. Cell Mol Life Sci.

[38] Surowiec R.K., Saldivar R., Rai R.K., Metzger C.E., Jacobson A.M., Allen M.R. (2023). Ex vivo exposure to calcitonin or raloxifene improves mechanical properties of diseased bone through non-cell mediated mechanisms. Bone.

[39] Amu G., Yang X., Luo H., Yu S., Zhang H., Tian Y. (2025). Machine learning-powered, high-affinity modification strategies for aptamers. Acta Materia Medica.

[40] Amu G., Zhang G., Jing N., Ma Y. (2024). Developing stapled aptamers with a constrained conformation for osteogenesis imperfect therapeutics. J Med Chem.

[41] Amu G., Ma Y., Yu S., Zhang H., Chen Z., Ni S. (2024). Unique quinoline orientations shape the modified aptamer to sclerostin for enhanced binding affinity and bone anabolic potential. Mol Ther Nucleic Acids.

[42] Zhang H., Yu S., Ni S., Gubu A., Ma Y., Zhang Y. (2023). A bimolecular modification strategy for developing long-lasting bone anabolic aptamer. Mol Ther Nucleic Acids.

[43] Manzano A., Herranz R., den Toom L.A., Te Slaa S., Borst G., Visser M. (2018). Novel, moon and Mars, partial gravity simulation paradigms and their effects on the balance between cell growth and cell proliferation during early plant development. NPJ Microgravity.

[44] Shen W., Shi P., Dong Q., Zhou X., Chen C., Sui X. (2023). Discovery of a novel dual-targeting D-peptide to block CD24/Siglec-10 and PD-1/PD-L1 interaction and synergize with radiotherapy for cancer immunotherapy. J Immunother Cancer.

[45] Sadowski M., Pankiewicz J., Scholtzova H., Ripellino J.A., Li Y., Schmidt S.D. (2004). A synthetic peptide blocking the apolipoprotein E/beta-amyloid binding mitigates beta-amyloid toxicity and fibril formation in vitro and reduces beta-amyloid plaques in transgenic mice. Am J Pathol.

[46] Qiu S., Divine G., Warner E., Rao S.D. (2020). Reference intervals for bone histomorphometric measurements based on data from healthy premenopausal women. Calcif Tissue Int.

[47] Meng Y., Zhang H., Li Y., Li Q., Zuo L. (2014). Effects of unfractionated heparin on renal osteodystrophy and vascular calcification in chronic kidney disease rats. Bone.

[48] Lyu S., Zhang C., Hou X., Wang A. (2022). Tag-based pull-down assay. Methods Mol Biol.

[49] Hirano S. (2012). Western blot analysis. Methods Mol Biol.

[50] Tian Q., Zhao S., Liu C. (2014). A solid-phase assay for studying direct binding of progranulin to TNFR and progranulin antagonism of TNF/TNFR interactions. Methods Mol Biol.

[51] Patterson K.R., Dalmau J., Lancaster E. (2018). Mechanisms of Caspr2 antibodies in autoimmune encephalitis and neuromyotonia. Ann Neurol.

[52] Jonkman J., Brown C.M. (2020). Tutorial: guidance for quantitative confocal microscopy. Nat Protoc.

[53] Grentzmann G., Ingram J.A., Kelly P.J., Gesteland R.F., Atkins J.F. (1998). A dual-luciferase reporter system for studying recoding signals. RNA.

[54] McNabb D.S., Reed R., Marciniak R.A. (2005). Dual luciferase assay system for rapid assessment of gene expression in Saccharomyces cerevisiae. Eukaryot Cell.

[55] Livak K.J., Schmittgen T.D. (2001). Analysis of relative gene expression data using real-time quantitative PCR and the 2(-Delta Delta C(T)) method. Methods.

[56] Zhao X.Y., Li W., Lv Z., Liu L., Tong M., Hai T. (2009). iPS cells produce viable mice through tetraploid complementation. Nature.

[57] Wang T., Bai J., Lu M., Huang C., Geng D., Chen G. (2022). Engineering immunomodulatory and osteoinductive implant surfaces via mussel adhesion-mediated ion coordination and molecular clicking. Nat Commun.

[58] Song R., Wang D., Zeng R., Wang J. (2017). Synergistic effects of fibroblast growth factor-2 and bone morphogenetic protein-2 on bone induction. Mol Med Rep.

[59] Gregory C.A., Gunn W.G., Peister A., Prockop D.J. (2004). An alizarin red-based assay of mineralization by adherent cells in culture: comparison with cetylpyridinium chloride extraction. Anal Biochem.

[60] Song C.Y., Guo Y., Chen F.Y., Liu W.G. (2022). Resveratrol promotes osteogenic differentiation of bone marrow-derived mesenchymal stem cells through miR-193a/SIRT7 axis. Calcif Tissue Int.

