# Supplementary material for: Blocking interaction of sclerostin loop3 with osteoblastic LRP4 counteracts bone loss without increasing arterial stiffness during mechanical unloading
Source: J Orthop Translat. 2026 Jun 8;59:101117. doi: 10.1016/j.jot.2026.101117 (PMC13263628; doi:10.1016/j.jot.2026.101117)
Supplement: Multimedia component 1 [file mmc1.pdf]

Supplementary Materials for

Blocking Interaction of Sclerostin Loop3 with Osteoblastic LRP4 Counteracts Bone Loss without Increasing Arterial Stiffness during Mechanical Unloading

This supplementary file includes:

Fig. S1. A schematic illustration summarizing the overall study design
Fig. S2. Either sclerostin loop3-specific deficiency in *Sost*$^{loop3-/-}$ mice or sclerostin loop3-specific inhibition by our tailor-made aptamer Apc001 counteracted unloading-induced bone formation reduction and bone loss without increasing arterial stiffness
Fig. S3. Bone phenotypes of *OB.Lrp4*$^{-/-}$ mice and the WT littermate under mechanical unloading (MUL) condition
Fig. S4. The mRNA/protein expression of *Sost*/sclerostin in osteocytes (MLO-Y4) and osteoblasts (MC3T3-E1) under MUL condition
Fig. S5. The blockade effects of Apc001/LRP4-Pep on binding between sclerostin and LRP4 under MUL condition
Fig. S6. Trabecular bone phenotypes at proximal tibia in *Lrp4m* mice, *Lrp4m/OB-Lrp4* mice and WT littermate under MUL condition
Fig. S7. Effects of LRP4-Pep on bone formation in WT mice under MUL condition
Table S1. Sequences of WT LRP4 and LRP4m

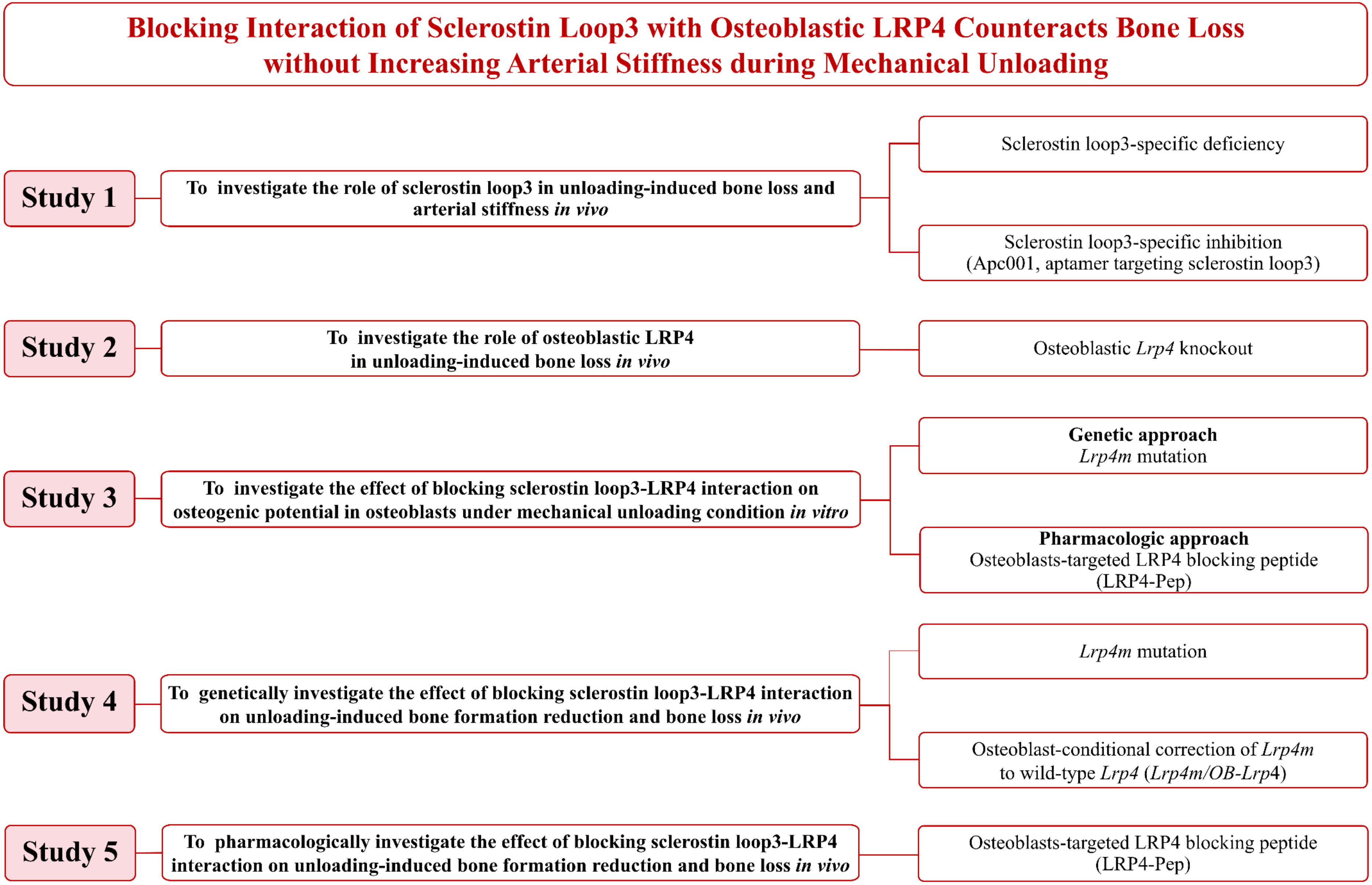

Fig. S1. A schematic illustration summarizing the overall study design

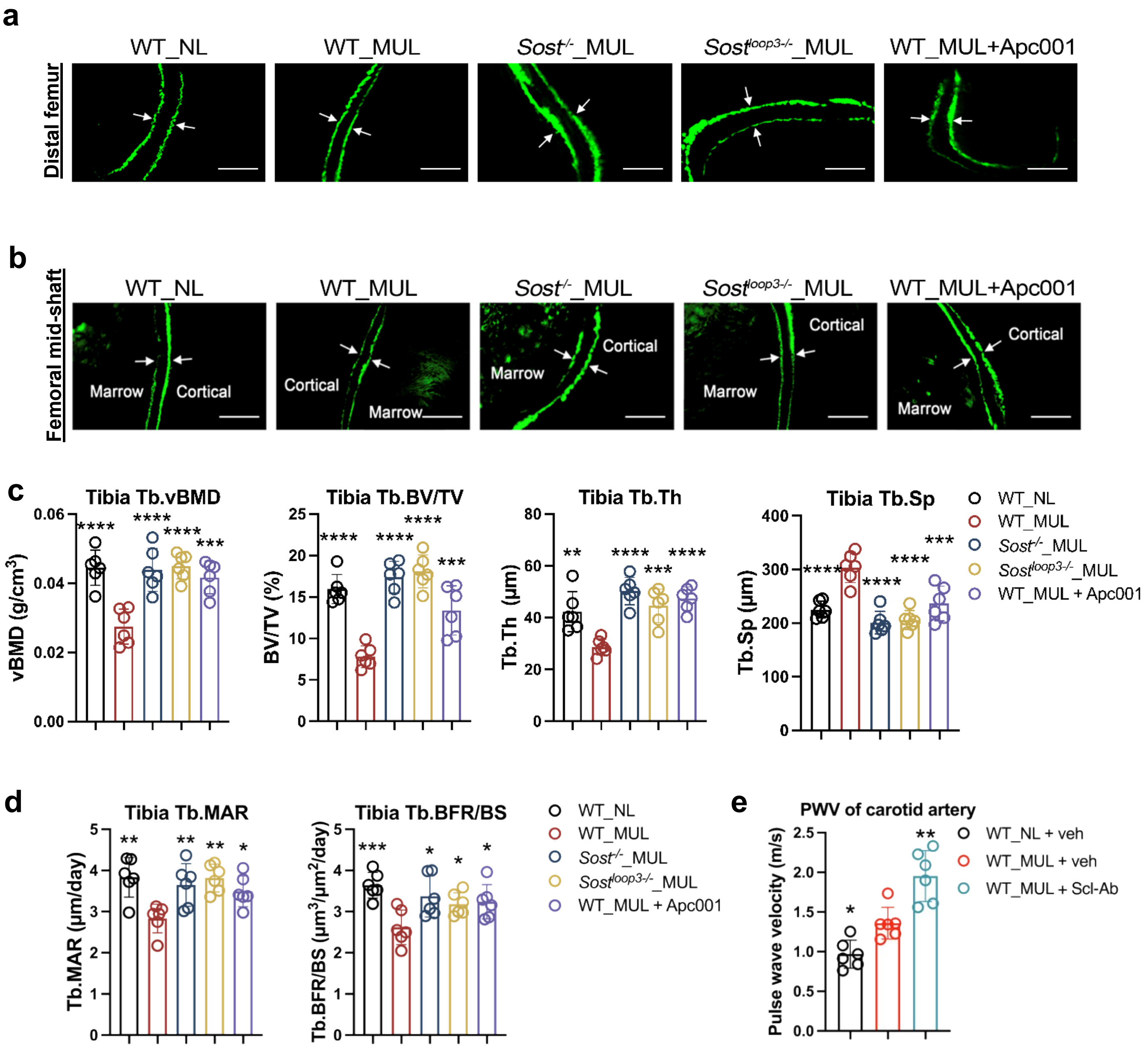

Fig. S2. Either sclerostin loop3-specific deficiency in *$Sost^{loop3-/-}$* mice or sclerostin loop3-specific inhibition by our tailor-made aptamer Apc001 counteracted unloading-induced bone formation reduction and bone loss without increasing arterial stiffness. (a) Representative fluorescent micrographs of the trabecular bone sections at the distal femur visualized by double calcein green labels. Arrows indicated the space between calcein green labeling. Scale bars, 20 μm. **(b)** Representative fluorescent micrographs of the cortical bone sections at the femoral mid-shaft visualized by double calcein green labels. Arrows indicated the space between calcein green labeling. Scale bars, 20 μm. **(c)** Bar charts of the structural parameters of Tb.vBMD, Tb.BV/TV, Tb.Th and Tb.Sp from *ex vivo* micro-CT examination at the proximal tibia. **(d)** Analysis of dynamic bone histomorphometric parameters of Tb.MAR and Tb.BFR/BS at the proximal tibia. **(a-d)** Data were expressed as mean ± standard deviation. n = 6 per group. ^{ns} *$P > 0.05$, * $P < 0.05$, ** $P < 0.01$, *** $P < 0.001$, **** $P < 0.0001$* for a comparison vs. WT_MUL by one-way ANOVA with Tukey's post-hoc test. **(e)** Sclerostin antibody (Scl-Ab) significantly elevated stiffness of carotid artery in hindlimb unloading mice. The bar chart of the carotid artery stiffness determined by Doppler ultrasound analysis. Data were expressed as mean ± standard deviation. N = 6 per group. ^{ns} *$P > 0.05$, * $P < 0.05$, ** $P < 0.01$, *** $P < 0.001$, **** $P < 0.0001$* for a comparison vs. WT_MUL + veh by one-way ANOVA with Tukey's post-hoc test. **NOTE**: NL: normal loading; MUL: mechanical unloading; Tb.vBMD: trabecular volumetric bone mineral density; Tb.BV/TV: trabecular bone volume per total volume; Tb.Th: trabecular thickness; Tb.Sp: trabecular spacing; Tb.MAR: trabecular bone mineral apposition rate; Tb.BFR/BS: trabecular bone formation rate; PWV: pulse wave velocity.

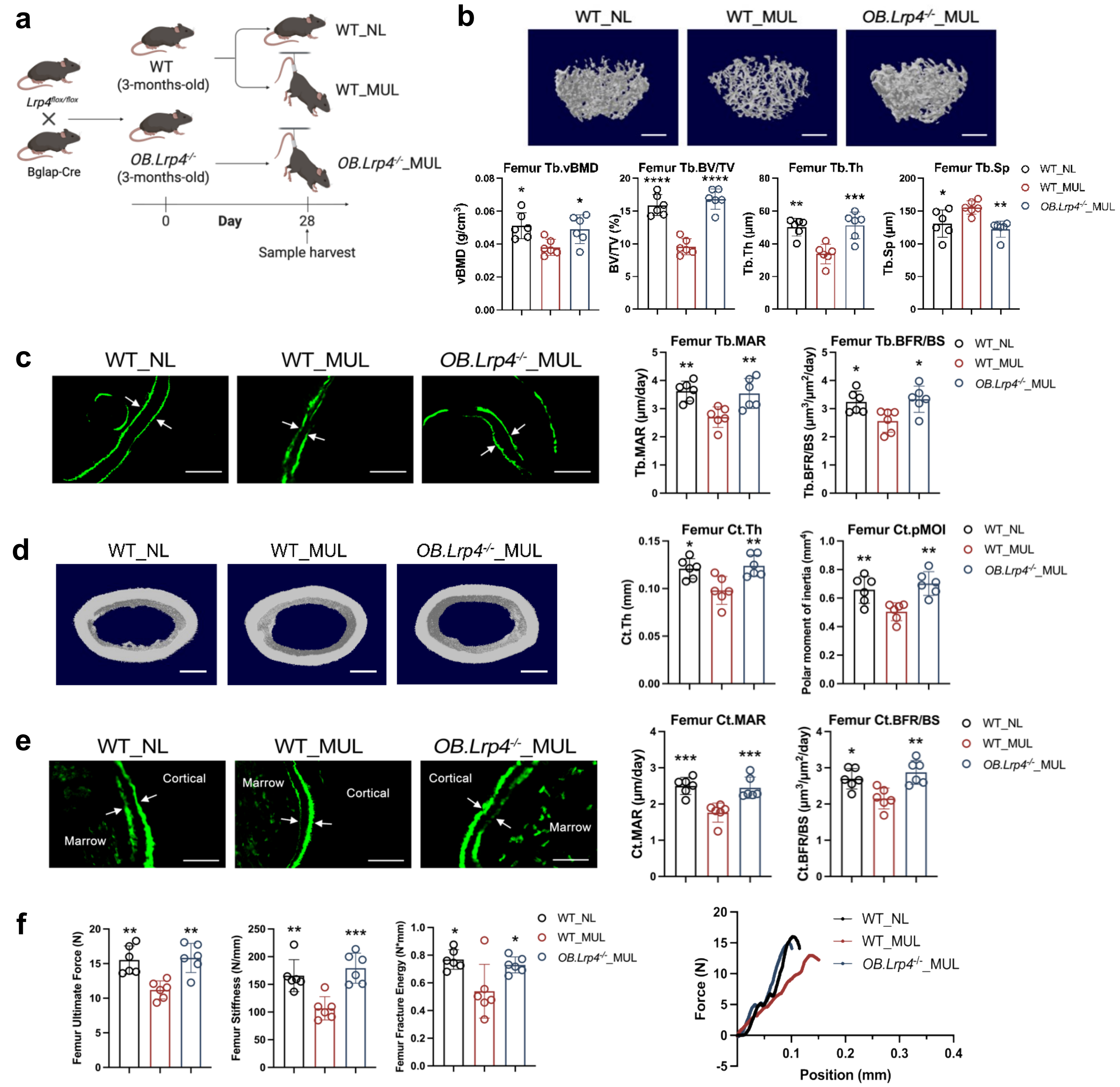

Fig. S3. Bone phenotypes of *OB.Lrp4$^{-/-}$* mice and the WT littermate under mechanical unloading (MUL) condition. (a) The diagram of experimental design. **(b)** Representative images showing three-dimensional trabecular bone microarchitecture by micro-CT reconstruction at distal femur of WT mice and *OB.Lrp4$^{-/-}$* mice (the upper panel). Scale bars, 200 μm. Bar charts of the structural parameters of Tb.vBMD, Tb.BV/TV, Tb.Th and Tb.Sp (the lower panel). **(c)** Representative fluorescent micrographs of trabecular bone sections at distal femur, visualized by double calcein green labels. Arrows indicated the space between calcein green labeling. Scale bars, 20 μm (the left panel). Dynamic bone histomorphometric parameters of Tb.MAR and Tb.BFR/BS at distal femur (the right panel). **(d)** Representative images showing three-dimensional cortical bone microarchitecture by micro-CT reconstruction at the femoral mid-shaft (the left panel). Scale bars, 200 μm. Bar charts of the structural parameters of Ct.Th and Ct.pMOI (the right panel). **(e)** Representative fluorescent micrographs of cortical bone sections at femoral mid-shaft visualized by double calcein green labels. Arrows indicated the space between calcein green labeling. Scale bars, 20 μm (the left panel). Analysis of dynamic bone histomorphometric parameters of Ct.MAR and Ct.BFR/BS (the right panel). **(f)** Bar charts of femur ultimate force (left), femur stiffness (middle left) and femur fracture energy (middle right). Representative curves showing the mechanical properties of the femora, determined by three-point bending test (right). Data were expressed as mean ± standard deviation. N = 6 per group. ^{ns} *$P > 0.05$, * $P < 0.05$, ** $P < 0.01$, *** $P < 0.001$, **** $P < 0.0001$* for a comparison vs. WT_MUL by one-way ANOVA with Tukey's post-hoc test. **NOTE**: NL: normal loading; MUL: mechanical unloading; OB: osteoblast; Tb.vBMD: trabecular volumetric bone mineral density; Tb.BV/TV: trabecular bone volume per total volume; Tb.Th: trabecular thickness; Tb.Sp: trabecular spacing; Tb.MAR: trabecular bone mineral apposition rate; Tb.BFR/BS: trabecular bone formation rate; Ct.Th: cortical thickness; Ct.pMOI: cortical polar moment of inertia; Ct.MAR: cortical bone mineral apposition rate; Ct.BFR/BS: cortical bone formation rate.

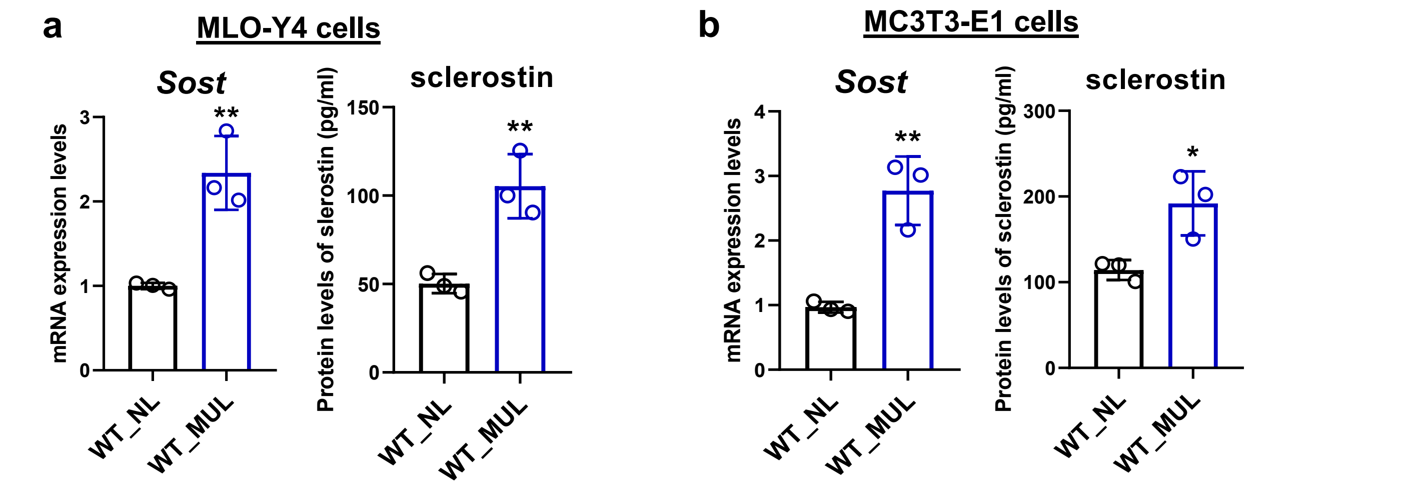

Fig. S4. The mRNA/protein expression of *Sost*/sclerostin in osteocytes (MLO-Y4) and osteoblasts (MC3T3-E1) under MUL condition. (a) The mRNA expression of *Sost* in MLO-Y4 cells, determined by RT-PCR (the left panel). The protein levels of sclerostin in MLO-Y4 cells (lysate and medium), determined by ELISA (the right panel). **(b)** The mRNA expression of *Sost* in MC3T3-E1 cells, determined by RT-PCR (the left panel). The protein levels of sclerostin in MC3T3-E1 cells (lysate and medium), determined by ELISA (the right panel). Data were expressed as mean ± standard deviation. N = 3 per group. ^{ns} *P > 0.05,* *P < 0.05, **P < 0.01, ***P < 0.001 and ****P < 0.0001 for intergroup comparison by unpaired t-test. **NOTE**: NL: normal loading; MUL: mechanical unloading.

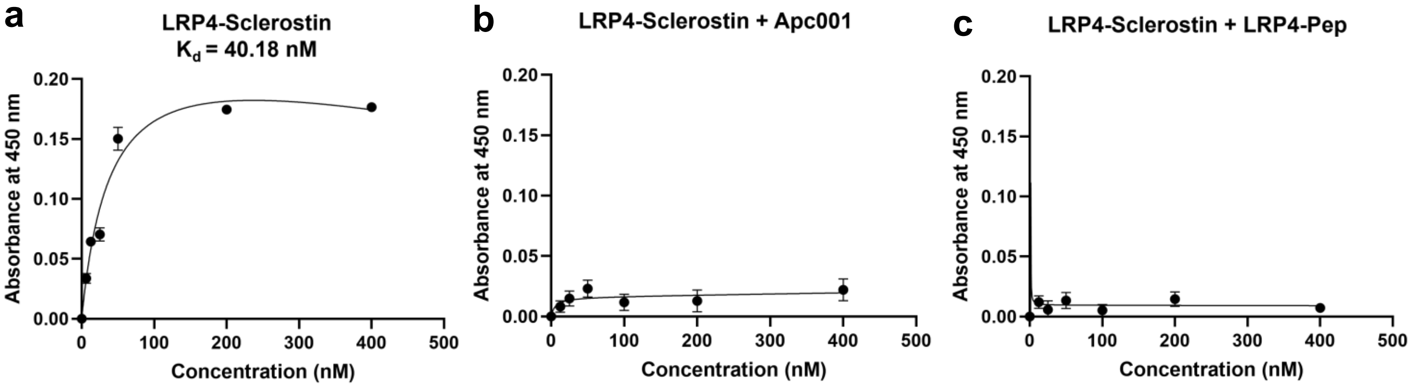

Fig. S5. The blockade effects of Apc001/LRP4-Pep on binding between sclerostin and LRP4 under MUL condition. (a) The binding affinity of sclerostin to LRP4, determined by biotinylated solid-phase binding assay. **(b)** The binding affinity of sclerostin to LRP4, after pre-incubation of sclerostin with Apc001. **(c)** The binding affinity of sclerostin to LRP4, after pre-incubation of sclerostin with LRP4-Pep.

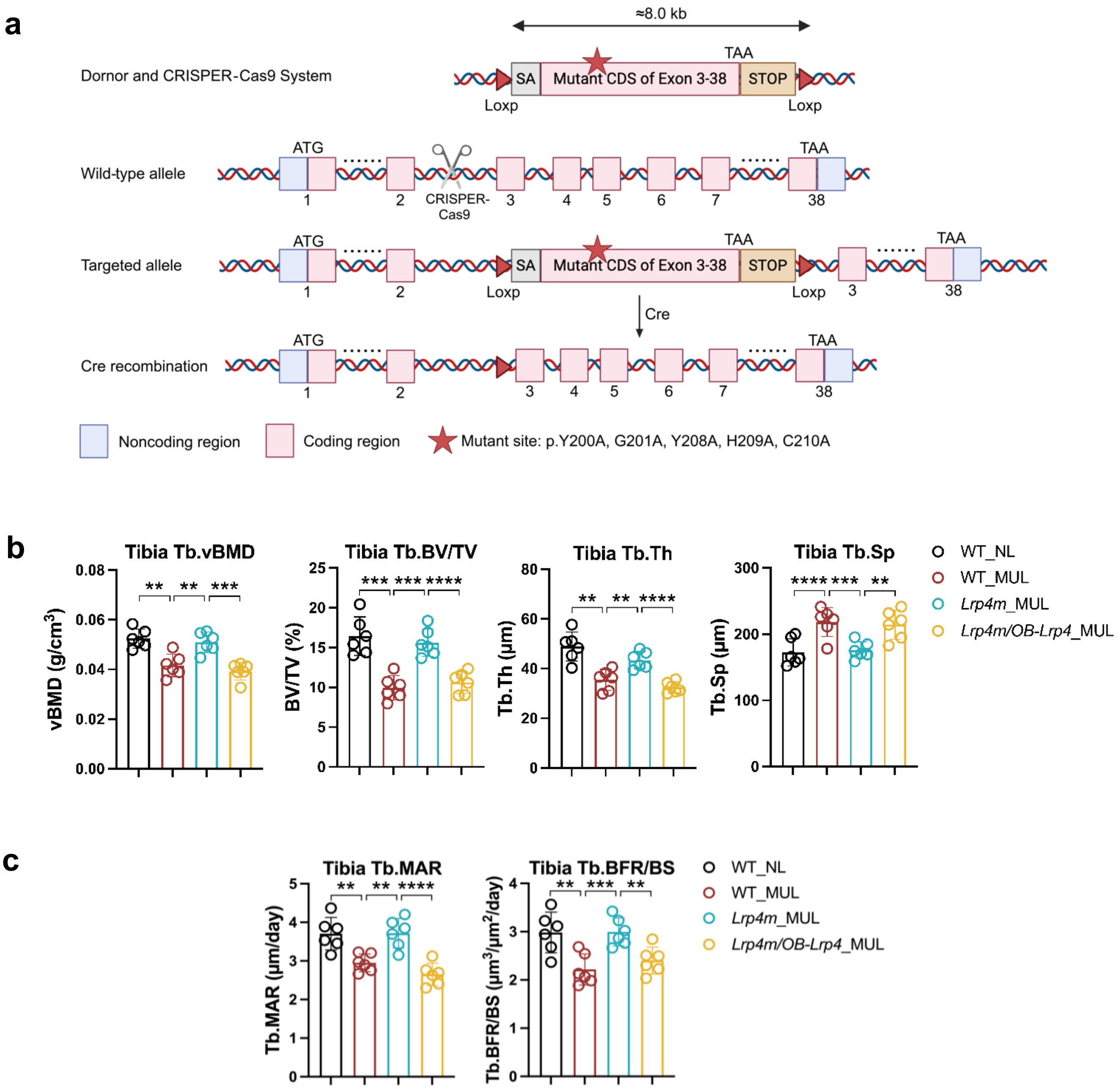

Fig. S6. Trabecular bone phenotypes at proximal tibia in *Lrp4m* mice, *Lrp4m/OB-Lrp4* mice and WT littermate under MUL condition. (a) The diagram for construction of *Lrp4m* mice and *Lrp4m/OB-Lrp4* mice. **(b)** Bar charts of the structural parameters of Tb.vBMD, Tb.BV/TV, Tb.Th and Tb.Sp from *ex vivo* micro-CT examination at the proximal tibia. **(c)** Dynamic bone histomorphometric parameters of Tb.MAR and Tb.BFR/BS at the proximal tibia. Data were expressed as mean ± standard deviation. N = 6 per group. ^{ns} *P > 0.05, *P* < 0.05, ***P* < 0.01, ****P* < 0.001 and *****P* < 0.0001 for intergroup comparison by unpaired t-test. **NOTE**: NL: normal loading; MUL: mechanical unloading; OB: osteoblast; Tb.vBMD: trabecular volumetric bone mineral density; Tb.BV/TV: trabecular bone volume per total volume; Tb.Th: trabecular thickness; Tb.Sp: trabecular spacing; Tb.MAR: trabecular bone mineral apposition rate; Tb.BFR/BS: trabecular bone formation rate.

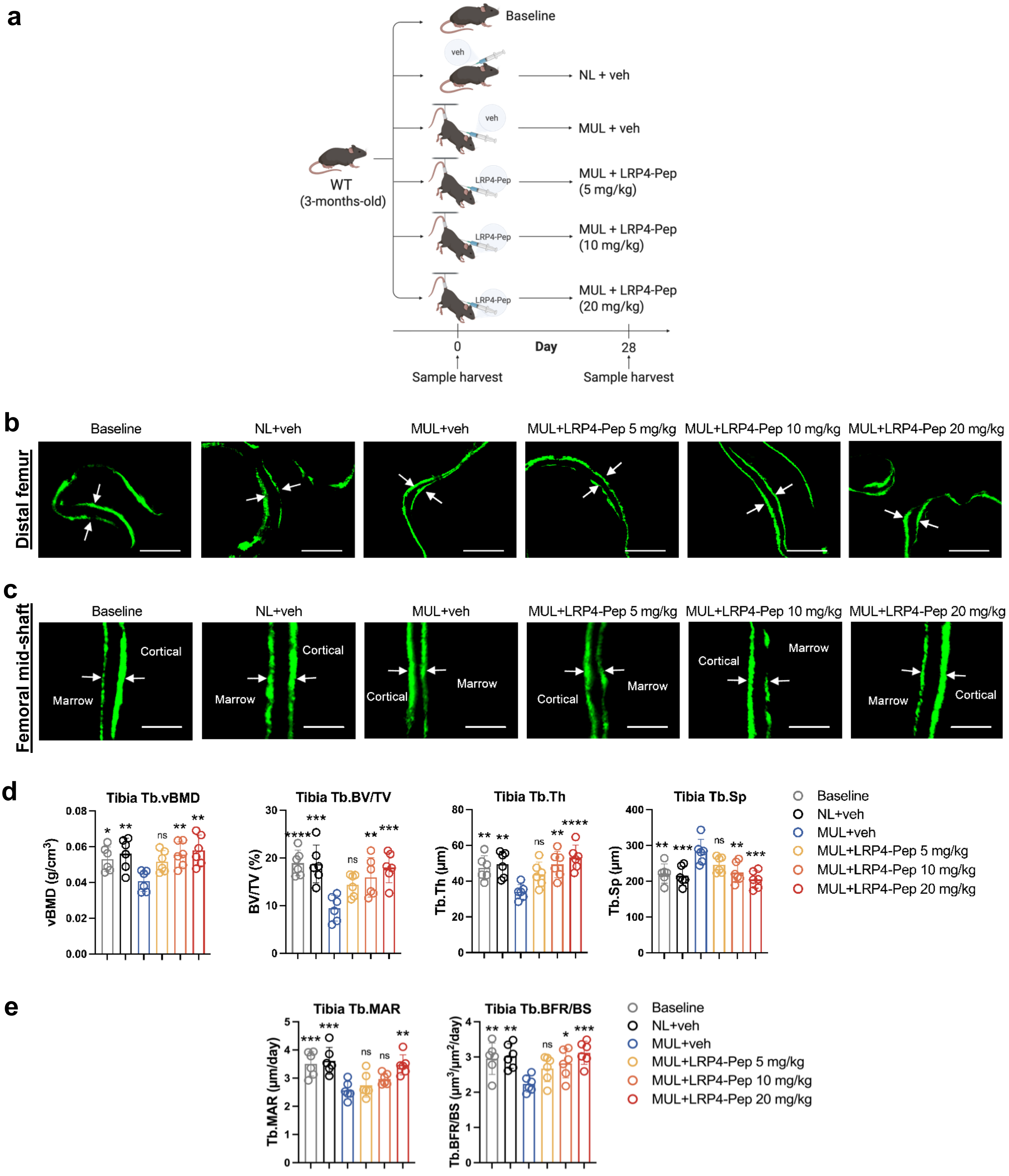

Fig. S7. Effects of LRP4-Pep on bone formation in WT mice under MUL condition. (a) The diagram of experimental design. **(b)** Representative fluorescent micrographs of trabecular bone sections at distal femur visualized by double calcein green labels. Arrows indicated the space between calcein green labeling. Scale bars, 20 μm. **(c)** Representative fluorescent micrographs of the cortical bone sections at the femoral mid-shaft visualized by double calcein green labels. Arrows indicated the space between calcein green labeling. Scale bars, 20 μm. **(d)** Bar charts of the structural parameters of Tb.vBMD, Tb.BV/TV, Tb.Th and Tb.Sp from *ex vivo* micro-CT examination at the proximal tibia. **(e)** Dynamic bone histomorphometric parameters of Tb.MAR and Tb.BFR/BS at the proximal tibia. N = 6 per group. ^{ns} *P > 0.05*, * *P < 0.05*, ** *P < 0.01*, *** *P < 0.001*, **** *P < 0.0001* for a comparison vs. MUL + veh by one-way ANOVA with Tukey's post-hoc test. **NOTE**: NL: normal loading; MUL: mechanical unloading; LRP4-Pep: LRP4 peptide tool; Tb.vBMD: trabecular volumetric bone mineral density; Tb.BV/TV: trabecular bone volume per total volume; Tb.Th: trabecular thickness; Tb.Sp: trabecular spacing; Tb.MAR: trabecular bone mineral apposition rate; Tb.BFR/BS: trabecular bone formation rate.

Table S1. Sequences of WT LRP4 and LRP4m

(Note: The sequences of LA5 domain within WT LRP4 and LRP4m were shown, the rest residues of LRP4 remained unchanged.)

Mutein ID	Sequence of LA5 domain within LRP4
WT LRP4	PCNLEEFQCAYGRCILDIYHCDGDDDCGDWSDESDCS
LRP4m	PCNLEEFQCAAARCILDIAAADGDDDCGDWSDESDCS
